# Distribution of natural radioactivity in different geological formations and their environmental risk assessment in Malaysia

**DOI:** 10.1007/s11356-024-33906-6

**Published:** 2024-06-20

**Authors:** Sheng Qin Seow, Prasanna Mohan Viswanathan, Dominique Dodge-Wan

**Affiliations:** Department of Applied Sciences, Faculty of Engineering and Science, Curtin University Malaysia, CDT 250, 98009 Miri, Sarawak Malaysia

**Keywords:** Radioactivity, Mineralogy, Gamma dose rate, Beta flux, NORM

## Abstract

Labuan, Miri, Kundasang and Raub regions of Malaysia have very different geological formations and settings that could result in different levels of natural radioactivity. Hence, this study determines the influence of different geological formations on radioactivity in these locations using field measurements, petrology and geochemistry. A total of 141 gamma dose rates and 227 beta flux measurements were collected using Polimaster survey meters (PM1405) in these four regions. The gamma dose rate values range from 0.37 to 0.05 µSv/h with a mean value of 0.11 µSv/h. Beta flux values range from 3.46 to 0.12 CPS with a mean value of 0.57 CPS. Mineralogy and elemental composition of the different rock types were analysed using thin-section petrography, XRD, ICP and *p*XRF methods. Felsic igneous rocks such as syenite and granite have higher natural radioactivity and contain more radionuclide-bearing minerals such as apatite, zircon, allanite, K-feldspar, titanite, muscovite and biotite. Metamorphic rocks have the second highest natural radioactivity and contain fewer radioactive minerals. The natural radioactivity of sedimentary rocks mostly depends on their clay content. The gamma dose rate maps show that igneous and metamorphic regions around Raub have higher radioactivity compared to the sedimentary-dominated regions around Miri and Labuan. Annual effective dose (AED) and excess lifetime cancer risk (ELCR) were calculated to evaluate the potential health risk for inhabitants of these regions. Labuan and Miri are considered to be safe zones with respect to natural radioactivity as the results show little to no risk for the public, compared with the Raub region, which is medium to high risk.

**Supplementary Information:**

The online version contains supplementary material available at 10.1007/s11356-024-33906-6.

## Introduction

Natural radiation exists everywhere on earth as it derives from cosmic sources, terrestrial sources, food ingested and building materials (Shahbazi-Gahrouei et al. 2013). Background radiation accounts for 80% of the total annual radiation dose for a typical human (Prihatiningsih et al. 2023). Geological materials are the major contributor to natural radioactivity (Kapanadze et al. 2021; Uyanik et al. 2022; Ofomola et al. 2023). The accumulation and distribution of radionuclides are often considered heterogeneous and depend on the abundance of certain minerals. They are incorporated as trace elements in crystal lattices during their formations (Chandrasekaran et al. 2015). Natural radioactivity of the rocks is from the primordial radionuclides such as potassium (^40^ K), thorium (Th), uranium (U) and other radioactive isotopes derived from the thorium and uranium decay series (Shahbazi-Gahrouei et al. 2013). All the isotopes of U and Th are radioactive, while ^40^ K is the only radioactive isotope that constitutes 0.0118% of potassium (Johnson 1991). The distribution and the concentration of primordial radionuclides, U, Th and ^40^ K determine the natural radioactivity of a geological unit, related to the minerals it contains (Chiozzi et al. 2007). U, Th decay series and ^40^ K are primary natural sources of ionising radiation in all types of geological units, such as igneous, metamorphic, sedimentary rocks and soils (Manjunatha et al. 2013; Uyanik et al. 2015; Lolila and Mazunga 2023; Lakshmi et al. 2024). The potential naturally occurring radioactive elements such as Ca, V, Ge, Se, Rb, Zr, Mo, Cd, In, Te, La, Ne, Sm, Eu, Gd, Lu, Hf, W, Rh, Pt and Bi either have a relatively longer half-life or are less abundant; thus, they are expected to have only minor impact on natural radiation (Asimov 1953). Notably, syenite shows distinctly high Ba, Rb and Sr content that is possibly related to mantle plume activity with high radioactivity in the rock (Ghani et al. 2002).

The influence of different geological units on natural radioactivity enables us to estimate the radiological risks that humans are exposed to and determine the background baseline values of natural radioactivity (Uyanik et al. 2013; Kazumasa et al. 2020; Ofomola et al. 2023). The inhalation of radon and thoron causes lung cancer by emitting high ionising alpha particles that interact with biological tissue in the lungs and damage the DNA of the lungs (Ahmad et al. 2017). Gamma, beta and alpha radiation are the standard forms of radiation produced by radionuclide decay. Natural occurring radioactive materials (NORM) associated with these forms of ionising radiation exhibit a relatively low level of natural radioactivity. They depend on the radionuclides’ concentration and decay rate usually present in minimal quantities (Missimer et al. 2019).

Numerous studies of natural radioactivity have been carried out on different geological units worldwide from 2005 to 2022 (Supplementary Table 1), and in Malaysia (Supplementary Table 2). Previous studies of natural radiation of geological units worldwide varied compared to the studies in Malaysia as they do not solely focus on the numerical value of radioactivity and they also used different methods to measure the radiation. Most of the researchers in Malaysia used the NaI survey meter and gamma spectrometry (HPGe) to measure the gamma dose rate and the activity concentration of radionuclides. Surface soil and the relevant basement rock are the main targets for their investigations. However, studies focusing on the natural radioactivity with respect to the geological formations are limited in Malaysia. It is also noted that limited radiation data is available in East Malaysia (Sarawak, Sabah on Borneo and Labuan) compared with Peninsular Malaysia. Hence, this study aims to establish the relationship between different geological units (igneous, sedimentary and metamorphic) and the natural radioactivity parameters (gamma dose rate and beta flux) in terms of petrography and geochemistry. This study also produced spatial maps of gamma radiation values and evaluated the risk to human health. The outcome of this study will enhance the natural radiation database for northern Borneo and increase the understanding of the role of geological units on natural radioactivity.

## Study locations

Raub in Pahang (Peninsular Malaysia), Miri in Sarawak (East Malaysia) and Kundasang and Labuan in Sabah (East Malaysia) have outcrops of various lithologies including igneous, metamorphic, meta-sedimentary and sedimentary origins as shown on the geological maps (Figs. 9, 10 and 11). This has resulted in different mineralogical assemblages which formed depending on the physical and chemical conditions during the geological history of the units (Mason and Berry 1968). Raub is known for its strategic geological location close to a major tectonic suture zone that features various rocks such as schist, amphibolite and granite, as well as conglomerates and other clastic rocks of which the oldest rocks are Lower Devonian (Khoo and Tan 1983). Meta-sedimentary, igneous and metamorphic rocks are located to the west of Raub, while sedimentary rocks are found to the east. The S-type Main Range Granite is commonly coarsely porphyritic microcline-alkali feldspar rock dated 207–203 Ma (Metcalfe 2000; Ghani et al. 2013). The granite intruded the Bentong-Raub suture zone and has a distinctive pattern of mineralisation. The slow cooling in a deep-seated environment allowed the alteration of alkali feldspars to microline, indicated by the absence of contact metamorphic aureoles during the isoclinal intrusion in folded phyllite and marble (Spiller 1998).

The geology of Labuan and Miri, with sedimentary rocks ranging from Oligocene to Upper Miocene, are relatively similar being both dominated by sandstone-shale and mudstone lithologies (Hutchison 2005). In addition, Labuan is often considered an extension of onshore formations in west Sabah, Brunei and northern Sarawak, with some common geological formations such as the Temburong, Setap Shale and Belait (Madon 1994). The Miri Formation outcrops within Miri City, and the Lambir Formation and Setap Shale are located to the south of Miri, while the Belait Formation outcrops in the north of Labuan.

The geology of the Kundasang area includes obducted Mesozoic ophiolite, Trusmadi and West-Crocker Formations of Eocene to Lower Miocene and the Neogene granitoid plutonism of Mt. Kinabalu (Hutchison 2005). Hence, this area hosts various geological formations, including basic, felsic igneous, meta-sedimentary and sedimentary rocks. Various igneous rock types, such as peridotites, serpentinite, gabbro and basalt, that are part of the ophiolitic suite are present in this region together with more recent meta-sedimentary and sedimentary rocks (Hall et al. 2009).

## Methodology

### Data acquisition

**Fig. 1:**
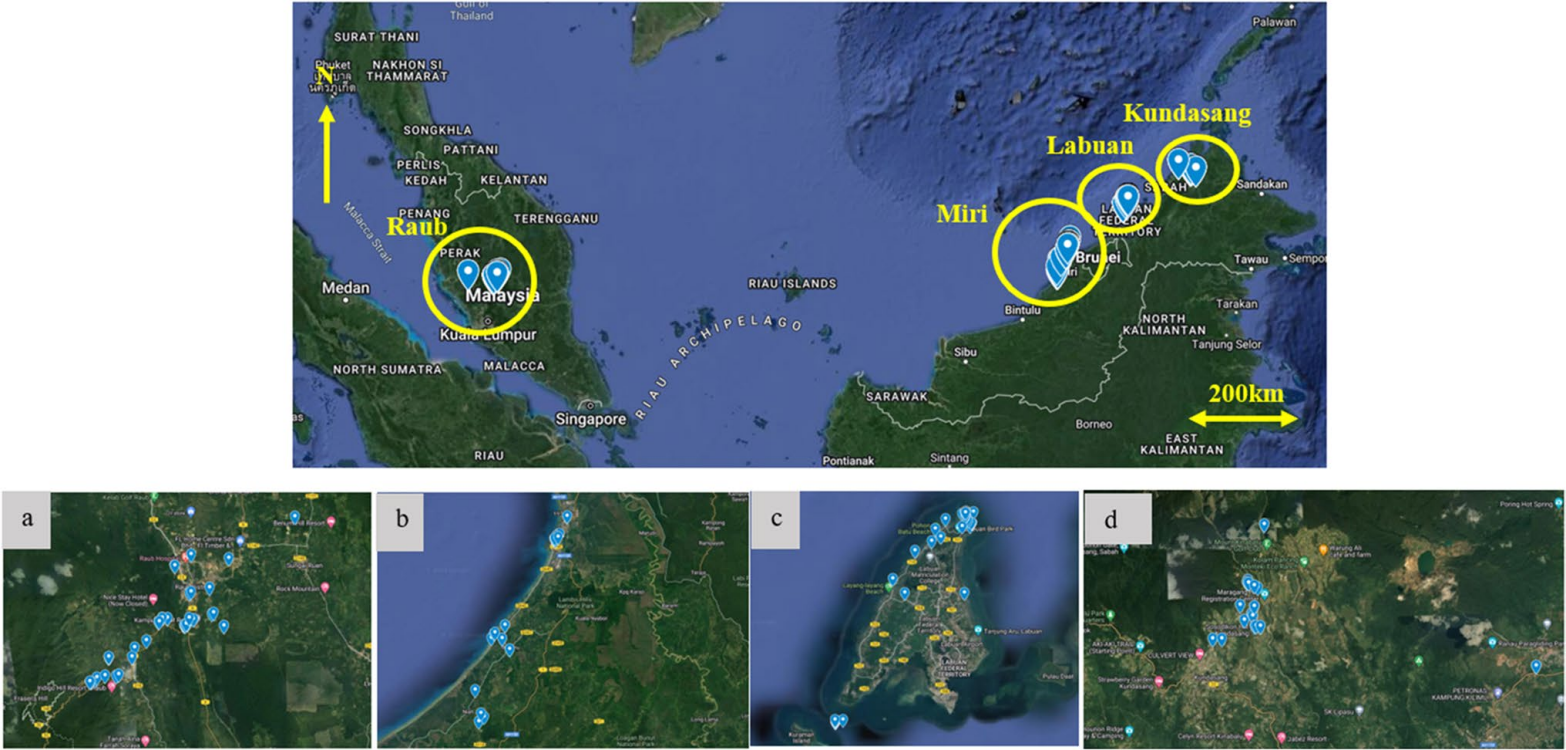
Natural radiation measurement locations ((**a**) Raub, (**b**) Miri, (**c**) Labuan, and (**d**) Kundasang)

In situ radiation measurements were conducted at and around four locations (Raub, Miri, Kundasang and Labuan) using Polimaster survey meters (PM1405). The Polimaster survey meters were used to measure both the ambient dose equivalent rate of gamma radiation and beta radiation flux density (beta plus gamma) from the sample surface. The gamma dose rate is measured in a unit micro sieve per hour (µSv/h), and the beta flux is measured in a unit count per second (CPS). The Polimaster survey meter was set on tripod 1 m above the ground surface for the gamma radiation measurement, while the meter was placed directly on the rock’s surface for the beta flux measurement. In addition, a 10% or less error percentage was maintained for every measurement. A total of 141 gamma dose rates and 227 beta flux readings were collected in the study areas (36 and 50 respectively in Labuan, 5 and 45 respectively in Kundasang, 65 and 72 respectively in Miri and 35 and 60 respectively in Raub). The location coordinates and the lithological details were also recorded at every data point. The locations of the data points are shown in Fig. 1.

### Mineralogy and geochemical analyses

In addition to the radiation ground surveys, four analytical methods were used to examine the mineral and elemental composition of selected samples from the respective study locations. The methods are thin-section petrography, X-ray diffraction (XRD), fusion ICP-OES/ICP-MS (ICP) and hand-held X-ray fluorescence (*p*XRF).

A total of 27 thin sections were made in selected rock samples, observed, photographed and analysed using Nikon Eclipse optical polarising microscope with a camera. Point counting was carried out to measure the percentage of each mineral in the thin sections (Supplementary Fig. 1). Each sample was named according to the proper classification of rock group such as Dott classification for sedimentary rock and QAP diagram for granitoid (Pettijohn et al. 1987; Le Bas and Streckeisen 1991; Schmid et al. 2007). Four samples were subjected to detailed laboratory analyses as given below.

- G1: syenite from Raub (igneous)
- S1: pyrite-graphite-quartz schist from Raub (metamorphic)
- M1: mudstone from Miri (sedimentary)
- MS: metasandstone from Raub (metasedimentary)

The samples were selected from rocks with significant high gamma dose rate and beta flux values. Detailed laboratory analyses included XRD and *p*XRF analysis. A sub-sample of 100 g was crushed and ground to powder of less than 63-micron grain size for three samples (G1, S1 and M1) were analysed for elemental composition in Activation Laboratories LTD in Canada. Code 4LITHO (11 +) Major Element Fusion ICP (WRA)/Trace Element Fusion ICP/MS (WRA4B2) packages were used for the geochemical analysis for the powdered samples. Another 30 g of powdered rock samples (G1, S1 and M1) were subjected to X-ray powder diffraction (XRD) analysis for mineral composition. XRD is a non-destructive, quick qualitative analysis of multi-component mixtures technique identified crystalline materials and solids from the x-ray beam reflection of the crystal lattice (Khan et al. 2020). In addition, an extra 10 g of each rock-powered sample (G1, S1, M1 and MS) was tested using a portable XRF analyser.

### Data analysis

Box and whisker charts of gamma dose rate and beta flux values were produced with all the acquired data to illustrate the maximum, minimum, normal range, interquartile range and average for each geological formation. Separate bar charts showing the gamma dose rate and beta flux for each study location were also plotted. Gamma dose rates in nGy/h were plotted in the frequency distribution histogram for each study location. In addition, skewness was calculated based on the curve in the frequency distribution histogram. The spatial maps of the gamma dose rate were also created using QGIS software.

## Results

### Mineralogical composition

**Fig. 2:**
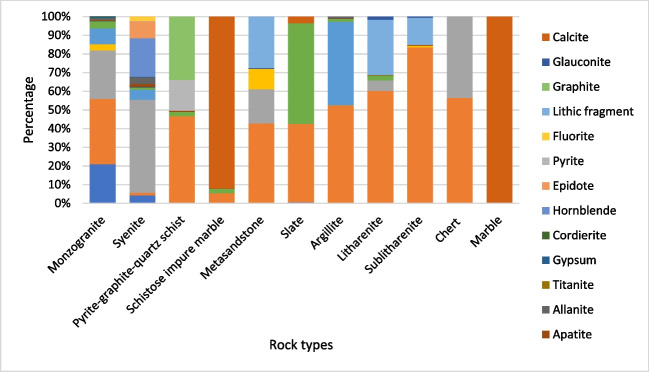
Mineral composition with weight % of collected samples in Raub

The samples collected in Raub region indicate diverse rock types such as monzogranite, syenite, schist, marble, meta-sandstone, argillite, chert and slate. Most of the analysed samples are rich in quartz except for a few rock types such as slate, marble, schistose impure marble and syenite. A total of 19 minerals were identified in the thin sections. Figure 2 shows the percentage of each mineral in selected samples from Raub region.

Monzogranite and syenite in Raub differ significantly in their major rock-forming minerals. Monzogranite is dominated by quartz, plagioclase and K-feldspar (82% in total), while syenite lacks quartz and consists predominantly of K-feldspar and hornblende (70% in total). Both rocks contain numerous minerals, including gypsum, allanite, fluorite, apatite, zircon, pyrite, cordierite and titanite. Other common secondary minerals such as muscovite, biotite and chlorite are present in both rocks (8.45 to 0.44%). Accessory minerals such as zircon, apatite and cordierite are often found within biotite, muscovite or chlorite crystals as euhedral mineral inclusions (Supplementary Fig. 1). Notably, epidote is quite common in syenite as it occurs as a secondary mineral resulting from the alteration of hornblende.

The metamorphic rocks in Raub, such as schist, meta-sandstone, marble and argillite, have different common mineral assemblages due to their different origins. Pyrite-graphite-quartz schist, metasandstone and argillite are dominated by quartz, ranging from 52.43 to 42.79%. Minor amounts of muscovite and biotite (< 11%) are present in the rocks, along with accessory minerals such as zircon, apatite and allanite (Supplementary Table 3). In addition, marbles are present with up to 100% calcite. Haile et al. (1977) stated that biotite, muscovite, sericite (alteration in plagioclase feldspar), chlorite and carbonaceous are common in the schist. Metcalfe (2000) also emphasised that the schists and phyllites in this area are strongly carbonaceous.

Sandstones from Miri were identified as sublitharenites and litharenite with grains consisting predominantly of quartz and lithic fragments (totalling up to 98.18%). Muscovite, biotite and glauconite are present in minor quantities (5.60 to 0.55%). Accessory minerals like zircon and apatite are also present in very minor amounts (< 0.2%) (Supplementary Table 3). Similar mineral composition was also observed by Siddiqui et al. (2017) and Hutchison (2005) in Miri and Belait Formations.

**Fig. 3:**
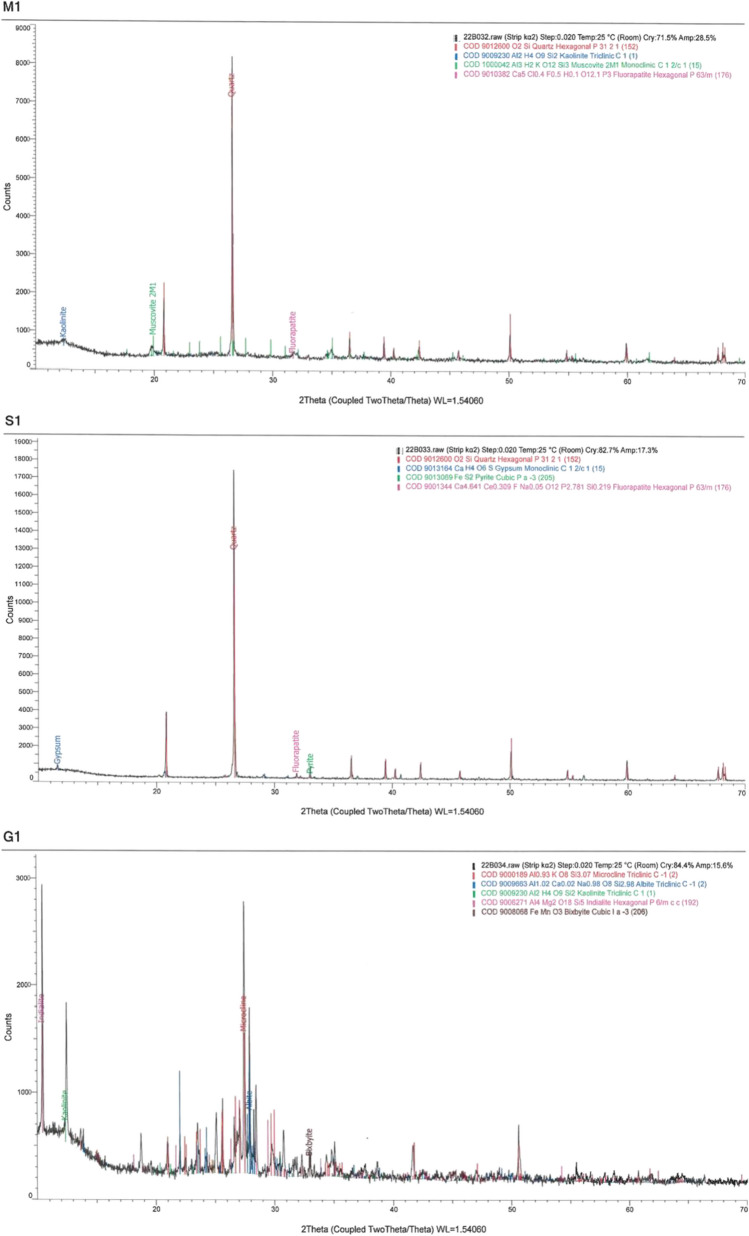
XRD results for samples. M1, mudstone; S1, pyrite-graphite-quartz schist; G1, syenite

From the XRD analysis, samples (M1, S1 and G1) showed different mineral compositions when matching the 2Ɵ peak pattern with respective minerals (Fig. 3). The XRD results for the pyrite-graphite-quartz schist (S1) match the observations from thin section and indicated quartz, pyrite, apatite and graphite as primary components, with minor amount of gypsum. The XRD results for the mudstone sample (M1) indicate quartz, kaolinite, muscovite and apatite as the key minerals. The XRD results for the syenite (G1) indicate the presence of microcline and albite with indialite, kaolinite and bixbyite.

### Elemental composition

**Fig. 4:**
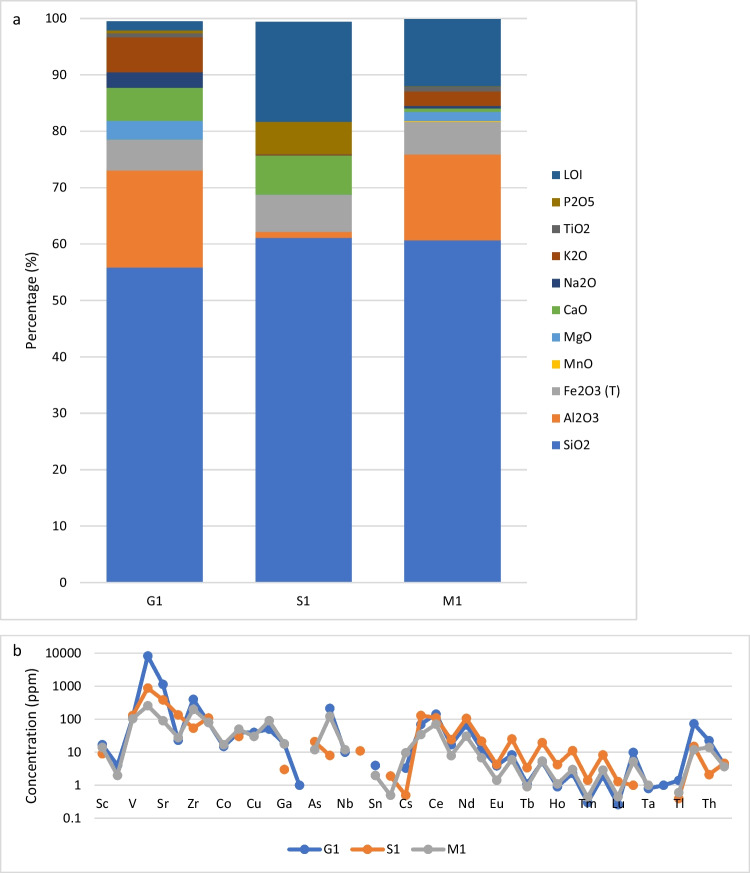
ICP results for major oxides (**a**) and trace element (**b**) concentration in the samples G1, S1 and M1

**Fig. 5:**
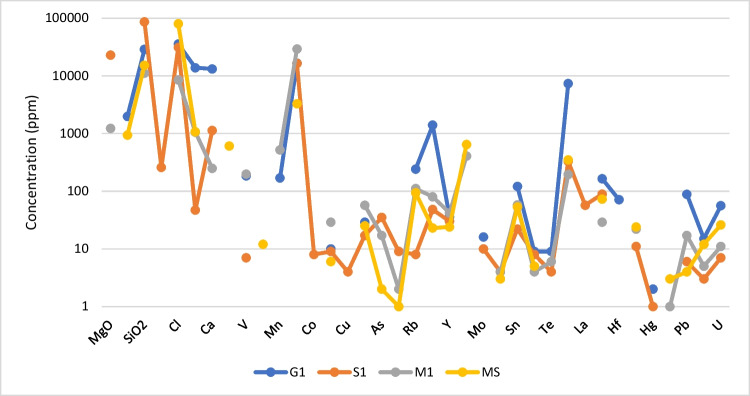
*p*XRF results for major oxides and trace element concentration in the samples G1, S1, M1 and MS

ICP and *p*XRF results indicate the major oxides/elements and the trace elements for G1, M1, S1 and MS samples (Figs. 4 and 5). ICP results show the syenite (G1) had the overall highest concentration of K_2_O (6.25%) and Th (22.6 ppm), whereas the schist (S1) had the lowest concentrations of K_2_O (0.18%) and Th (2.1 ppm), with the highest U content (4.6 ppm). The schist (S1) has a higher concentration of rare earth elements (REE) (Sc, Y, La, Ce, Pr, Nd, Pm, Sm, Eu, Gd, Tb, Dy, Ho, Er, Tm, Yb, Lu) compared to other samples, and REE content often associated with radionuclides.

*p*XRF results show the syenite (G1) had the highest K_2_O, U and Th concentrations (13,808 ppm, 56 ppm and 15 ppm, respectively). The other samples, metasandstone (MS), mudstone (M1) and schist (S1), contained less. Both results (ICP and *p*XRF) show similar trends for elements Ba, Sr, Rb and Zr with the syenite (G1) having distinctively higher values compared to other rock types.

### Natural radioactivity

**Fig. 6:**
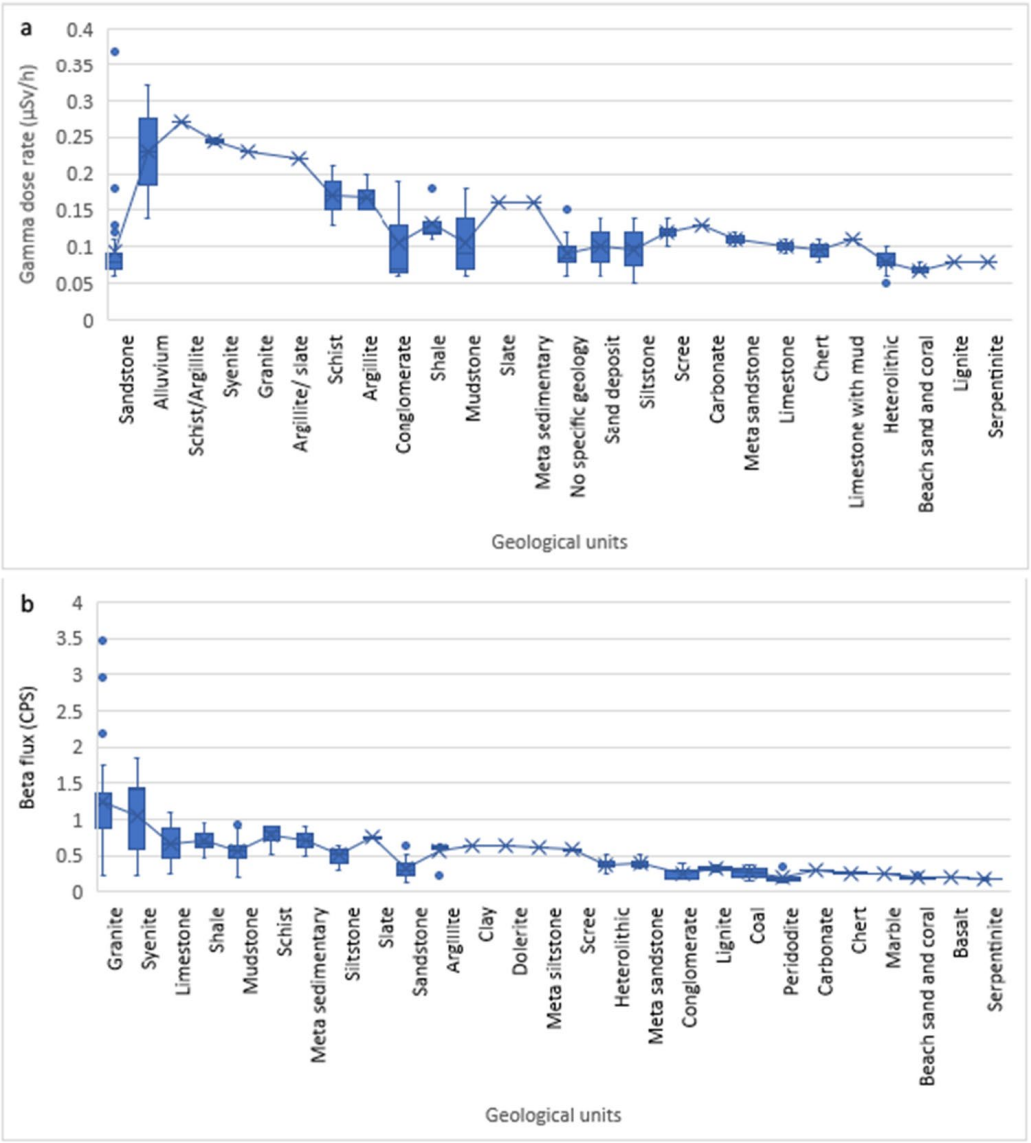
Box and whisker plot for all the data points at all the study areas. **a** Gamma dose rate and **b** beta flux (the dots represent the outliers)

The gamma dose rates and beta flux values obtained have been sorted according to the corresponding geological units. The results for gamma dose rate at Labuan, Raub and Miri have a skewness of 0.15, 0.38 and 0.12, respectively, indicating they are normally distributed data (Supplementary Fig. 2). Thirty-five geological units were identified covering all the data collected in the four areas. Figure 6 shows the overall natural radioactivity data with a mean line (average) in all areas. Gamma dose rate had a mean value of 0.11 µSv/h and ranged from 0.05 to 0.37 µSv/h, while beta flux had a mean value of 0.57 CPS and ranged from 0.12 to 3.46 CPS. Overall, syenite and granite show the highest beta flux, followed by metamorphic and sedimentary rocks (slate, metasedimentary, schist, mudstones, sandstones, marble, chert, mafic igneous rock and sediment). The gamma dose rate does not reflect the natural radioactivity of individual rock types, as other factors could influence the radiation. The results from each location are presented here below:

### Raub

Nineteen geological units were identified in the Raub area during the data acquisition. Overall, among the four study areas, the Raub area had the highest mean gamma value (0.176 µSv/h) and the second highest mean beta flux value of 0.774 CPS. Supplementary Fig. 3 shows the Raub area’s gamma dose rate and beta flux results. The gamma dose rate in the Raub area ranged from 0.11 to 0.32 µSv/h, and the beta flux ranged from 0.23 to 1.84 CPS. The Raub area had higher readings in gamma dose rate and beta flux than the other three areas. Hard rocks such as syenite, granite and schist are expected to have higher values for both gamma dose rate and beta flux compared to sedimentary rocks like sandstone and mudstone. Beta flux in the Raub area is more variable across different rock types than in other areas. However, part of the syenite and granite data points (right side of the graph, Supplementary Fig. 3) from beta flux showed some diverse values that differ from the left side of the graph (Supplementary Fig. 3).

### Miri and Labuan

The study areas in Miri and Labuan appear to have similar natural radioactivity levels, and this is consistent with their similar geological settings. Supplementary Fig. 4 shows a bar chart of the natural radioactivity for the Miri area with a mean gamma dose rate value of 0.092 µSv/h and mean beta flux value of 0.392 CPS. During the data acquisition, nine types of lithologies were identified and recorded in the Miri area. The gamma dose rate values obtained in the Miri area are very consistent as the majority of data points are between 0.06 and 0.14 µSv/h which is a narrow range of ± 0.08 µSv/h in comparison to other study areas where more variability was noted. Beta flux values in the Miri area ranged from 0.16 to 0.77 CPS. When comparing the lithologies, mudstone units have higher values (from 0.37 to 0.77 CPS) compared to chert, sandstone and lignite with beta flux between lower values (0.16 and 0.36 CPS).

Supplementary Fig. 5 shows the result of the gamma dose rate and beta flux in the Labuan area. The results indicate for the Labuan area a mean gamma dose rate value of 0.081 µSv/h and mean beta flux value of 0.326 CPS. Eight lithological units were identified and recorded in the Labuan area. The results are similar to those obtained in the Miri area. The gamma dose rate values range from 0.05 to 0.16 µSv/h, and beta flux values range from 0.14 to 0.67 CPS. As in the Miri area, the fine-grained clastic rocks such as mudstone and siltstone generally have higher beta flux values (0.21 to 0.67 CPS) than coarser rocks such as sandstone, conglomerate, beach sand and coal (0.14 to 0.39 CPS).

### Kundasang

Supplementary Fig. 6 shows the gamma dose rate and beta flux in the Kundasang area. The results show that of the four study areas, the Kundasang area had the highest mean beta flux value of 0.836 CPS (45 measurements) with values ranging from 0.12 to 3.46 CPS. Only five gamma dose rate measurements were recorded in this area with a mean value of 0.11 µSv/h which is slightly higher than Miri and Labuan but lower than Raub. Felsic and mafic igneous rocks in this area were found to have variable beta flux values: the felsic rocks (such as granite) have higher values, up to 3.46 CPS, and mafic igneous rocks (such as serpentinite, dolerite and peridotite) have the lower values. Metasedimentary and sedimentary rock did not show many differences compared to other areas.

## Discussion

### Radionuclide-bearing minerals

In this study, the occurrences of zircon, allanite and apatite are the significant radiation contributors besides potassium minerals. A similar observation was made by Kohman and Saito (1954) who stated that the natural radioactivity of igneous rocks is concentrated within the accessory minerals such as zircon, allanite, sphene, uraninite, thorite, apatite and monazite. Other minerals such as epidote, quartz, feldspar, hornblende, pyroxene, magnetite and mica may have minor or trace contributions to radioactivity even though they constitute a significant proportion of the rocks.

The presence of zircon, a widely distributed accessory mineral, may contribute to the radioactivity of rocks containing it, but its occurrence is less common than allanite and apatite. Selby (2007) stated that U and Th are incorporated in the crystal lattice of all zircons (0.025–0.035 wt% U and 0.01–0.02 wt% Th). They were fused into the zircon structure during the crystallisation of zircon in a molten state. U and Th can substitute Zr in the structure due to their similar atomic size (Mason and Berry 1968). In addition, hafnium (Hf) is commonly present in zircon at around 1 wt%, and one of the isotopes of hafnium (^174^Hf) is considered radioactive. One expected evidence for zircon’s radioactive characteristic is pleochroic haloes caused by radiation damage if the zircon is enclosed by biotite, amphibole, or other coloured silicates (Deer et al. 2013).

In this study, allanite was found to be present (3.7% to 0.84%) in syenite, argillite and monzogranite. Hence, it could be one of the most significant contributors to radiation in these rocks as it has higher U and Th contents than other accessory minerals. Allanite is an accessory mineral commonly found in syenite, granites, diorites and their metamorphic derivatives (Gribble 1988). Biotite, hornblende or other mafic silicate minerals are often found along with allanite (Nesse 2000). Deer et al. (2013) stated that most allanites consist of up to 5 wt% ThO_2_ and 0.5 wt% U_3_O_8_ (4.39 wt% Th, 0.43 wt% U) causing the metamictisation of allanites and brown haloes in the surrounding rocks. Th and U could present significant amounts in allanite by substituting the Ca in the crystal structure (Nesse 2000).

Apatite also is considered a possible major contributor of radioactivity in sedimentary and sedimentary-derived metamorphic rocks, in which other radioactive accessory minerals are not present. Most of the samples analysed in this study contain 1.3 to 0.15% apatite. However, no apatite was found in the marble, slate, chert, metasandstone and sublitharenite. Apatite commonly contains trace amounts of uranium, vanadium, manganese and iron (Nesse 2000). Vásconez-Maza et al. (2022) reported that sedimentary origin apatite is more radioactive than igneous origin apatite, due to the presence of ^40^ K in sedimentary origin apatite. Apatite of igneous origin could contain 0.001 to 0.1 wt% of U, while sedimentary marine apatite can contain as much as 0.005 to 0.2 wt% of U. U can substitute for calcium in the apatite crystal structure as both elements have identical ionic radii (Altschuler et al. 1957). A study by Altschuler et al. (1957) shows Th in igneous apatite could be three or four times more abundant than U if it is enriched with rare earth elements. However, due to the phosphorite lack of Th, U will be the primary radioactivity contributor. In addition, U can be enriched or postdepositionally leached from apatite, which dramatically modifies the radioactivity of apatite depending on the environment (Altschuler et al. 1957).

K-feldspar, biotite, muscovite, kaolinite and glauconite are the major potassium-bearing minerals present in various percentages in the samples analysed in this study. K-feldspar is abundant in syenite, monzogranite and chert and may constitute up to 49.9% of these rocks. Micas are also present in every rock at percentages ranging from trace to 53.9%. In this study, most of the count-per-second rate of beta flux radioactivity came from the ^40^ K, considered more significant than U or Th in almost all rocks except carbonate rocks (Johnson 1991). Glauconite is only present in sandstones ranging from trace to less than 2%. Due to its abundance, it is considered to have similar radioactivity as accessory minerals. Slate in this study was composed of 53.9% muscovite, which had a high radiation value and could contribute significant radioactivity to the rock. Potassium is generally sourced from many minerals, which can be categorised into three groups which are clay minerals (e.g. illite and kaolinite), rock-forming minerals (e.g. K-feldspar and micas) and evaporates (e.g. sylvite and carnallite) (Schön 2011). Radioactive isotope (^40^ K) and stable isotope (^39^ K) are the naturally occurring forms of potassium, at concentrations of 0.012% and 99.988%, respectively (Garner et al. 1975). Schön (2011) summarised the K, U and Th content of rock-forming minerals, of which K-feldspar has the highest potassium content, up to 16%.

Granger and Raup (1925) reported that fine-grained graphite from the Rainbow deposit in the USA was found to be a uranium-bearing mineral. They suggested that some of U is associated with graphite and carbon due to the strongly radioactive mineralogic separation of graphite. In this study, pyrite-graphite-quartz had a high portion of graphite, occupying 33.89% of the total minerals. This example could explain the high radiation noted in schist compared to other rock types, as there are no other radionuclide-bearing minerals except apatite and muscovite present in these samples. Other minerals, such as epidote and titanite, that only occur in particular rocks are responsible for these rocks’ radioactivity, but these minerals’ overall radioactivity cannot be justified due to a lack of comparison in samples. Further in-depth studies need to be done on graphite as the radioactivity properties of graphite remain less well known.

### Correlation between radionuclides and radiation

**Fig. 7:**
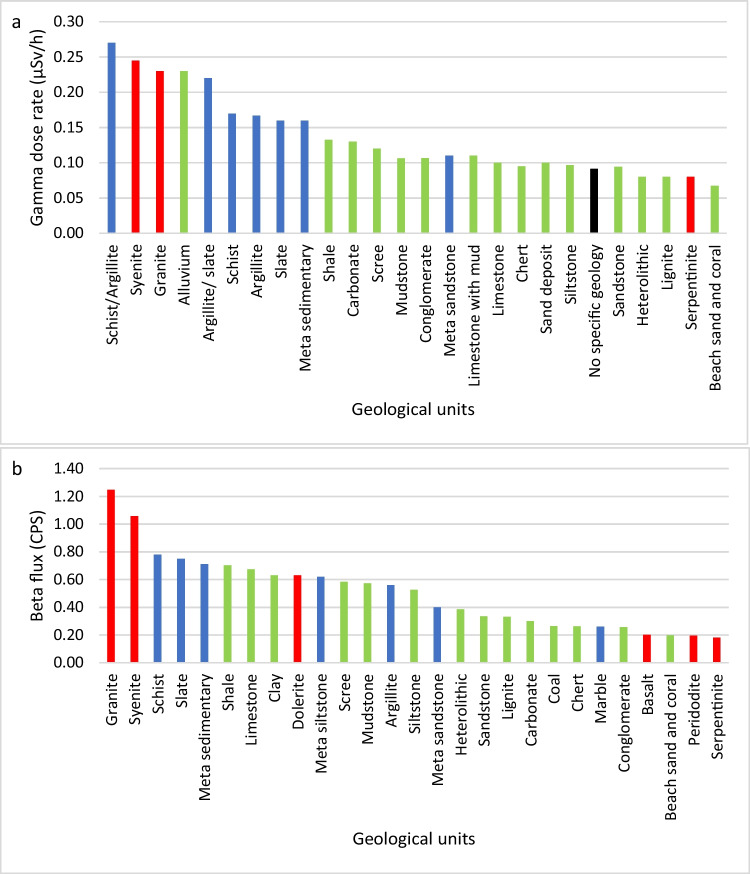
Average value for each geological unit in all the study areas. **a** Gamma dose rate and **b** beta flux. Igneous rock (red), metamorphic rock (blue), sediment and sedimentary rock (green)

Figure 7a shows the average gamma dose rate values for each lithology across the four study areas, arranged in order of decreasing gamma and grouped by colour coding into igneous, metamorphic and sedimentary rock types. Felsic igneous and metamorphic rocks such as syenite, granite, schist, slate and metasedimentary rocks have higher average gamma dose rate values than mafic igneous rocks, sedimentary rocks and sediments. It is interesting to observe that the alluvium shows higher gamma dose rate values and is a dominant material in the coastal regions of Miri and Labuan. Alluvium consists of unconsolidated materials including clay, which was derived from the older sedimentary rocks (i.e. shale) in these regions. This leads to the higher gamma dose rate values (DaPelo et al. 2024).

**Fig. 8:**
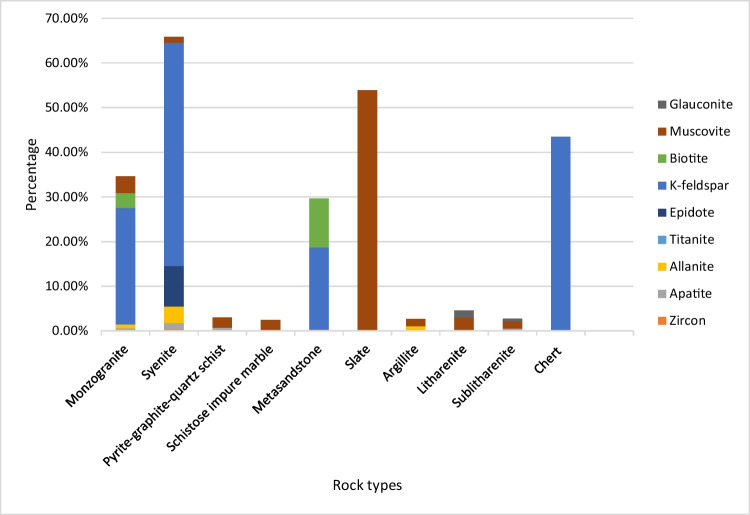
Radioactive minerals in each rock type

The average beta flux for each rock type, given in Fig. 7b, has a similar pattern with felsic igneous and metamorphic rocks showing higher average values and sedimentary rocks showing lower average values. Figure 8 illustrates the percentage of radionuclide-bearing minerals in the rock types tested, and these may contribute to the rocks’ natural radioactivity. By comparing these percentages with the radiation values, igneous rocks such as syenite and granite show higher average beta flux followed by metamorphic rocks, sedimentary rocks, sandy sediments and mafic igneous rock. Here, below, they are discussed in the following order: igneous, metamorphic and sedimentary rocks.

Monzogranite and syenite with higher percentages of radioactive minerals (especially accessory minerals like zircon, allanite and apatite) and radionuclide contents (U and Th) have the highest average beta flux (1.25 CPS and 1.06 CPS, respectively) compared to other rocks. Generally, igneous rocks have the ability to accumulate large incompatible nuclei in different rock-forming minerals and accessory minerals which leads to granitoid felsic igneous rocks having potential for radioactive isotopes like U, Th and ^40^ K (Younis et al. 2022). The high abundance of rock-forming minerals in granitoid such as feldspar and micas contributes to a high level of ^40^ K and a higher proportion of radionuclide-bearing minerals compared to other geological units, and this leads to igneous rocks having significantly higher natural radioactivity (Manjunatha et al. 2013). In this study, mafic igneous rocks were found to have very low natural radioactivity (0.18 to 0.63 CPS) mainly because of their lack of potassium-bearing minerals and predominantly low or non-radioactive minerals such as pyroxene, olivine and plagioclase feldspars (Schön 2011).

Metamorphic rocks observed in this study included schist, argillite, slate, metasedimentary rocks and marble with different mineral compositions. From this study, slate, metasandstone and argillite have relatively more radioactive minerals than marble and sandstone (litharenites). Schist and slate have the highest average beta flux values (0.78CPS and 0.75 CPS, respectively) among the analysed metamorphic rocks, which could be related to a higher apatite and muscovite content. However, the *p*XRF and XRD result for pyrite-graphite-quartz schist does not show a significantly higher percentage of radioactive minerals and radionuclides than mudstone, which is probably due to the heterogeneity of accessory minerals and radioactive isotopes. Abdulqader et al. (2018) also observed that the schist sample with higher graphitic content also contains more uranium. However, quartz in metamorphic rocks can show insignificant radioactivity (Manjunatha et al. 2013). In this study, metamorphic rocks such as argillite and metasandstone, including metasedimentary rocks, were found to contain a considerable amount of radionuclide-bearing minerals and potassium minerals that contributed to medium to high radioactivity (0.4 to 0.71 CPS on average). Schön (2011) stated that the U and Th content is depleted with increasing metamorphism due to dehydration, while potassium is less affected by the process. The marble that possessed calcite as the only primary mineral contains no uranium, which is consistent with the low radioactivity (beta flux of 0.26 CPS), but certain classes of carbonate rock, not observed in this study, could potentially contain above-average amounts of uranium (Bell 1963).

The sedimentary rocks analysed in this study show varied beta flux values for mudstones/shales and siltstones (0.7 to 0.53 CPS on average), which have higher values than sandstones (0.33 CPS on average). Mudstones/shales contain apatite and muscovite, and a certain amount of U and Th concentration could explain the higher natural radioactivity recorded for mudstones/shales compared to sandstones. This may be due to the presence of potassium, thorium and uranium favour illite (clay minerals), shales and phosphates, respectively, as reported by Schön (2011). However, the natural radioactivity of sedimentary rocks may also be affected by the oxidation process during weathering and erosion which may mobilise uranium and in turn which can result in either its enrichment or depletion (Alnour et al. 2014). Generally, sedimentary rocks with higher clay contents tend to present higher radioactivity as observed in this study. The same applied to limestones, sandstones, heterolithic rocks (sand-mud), other sediments and siltstones.

### Spatial variation of TGDR

**Fig. 9:**
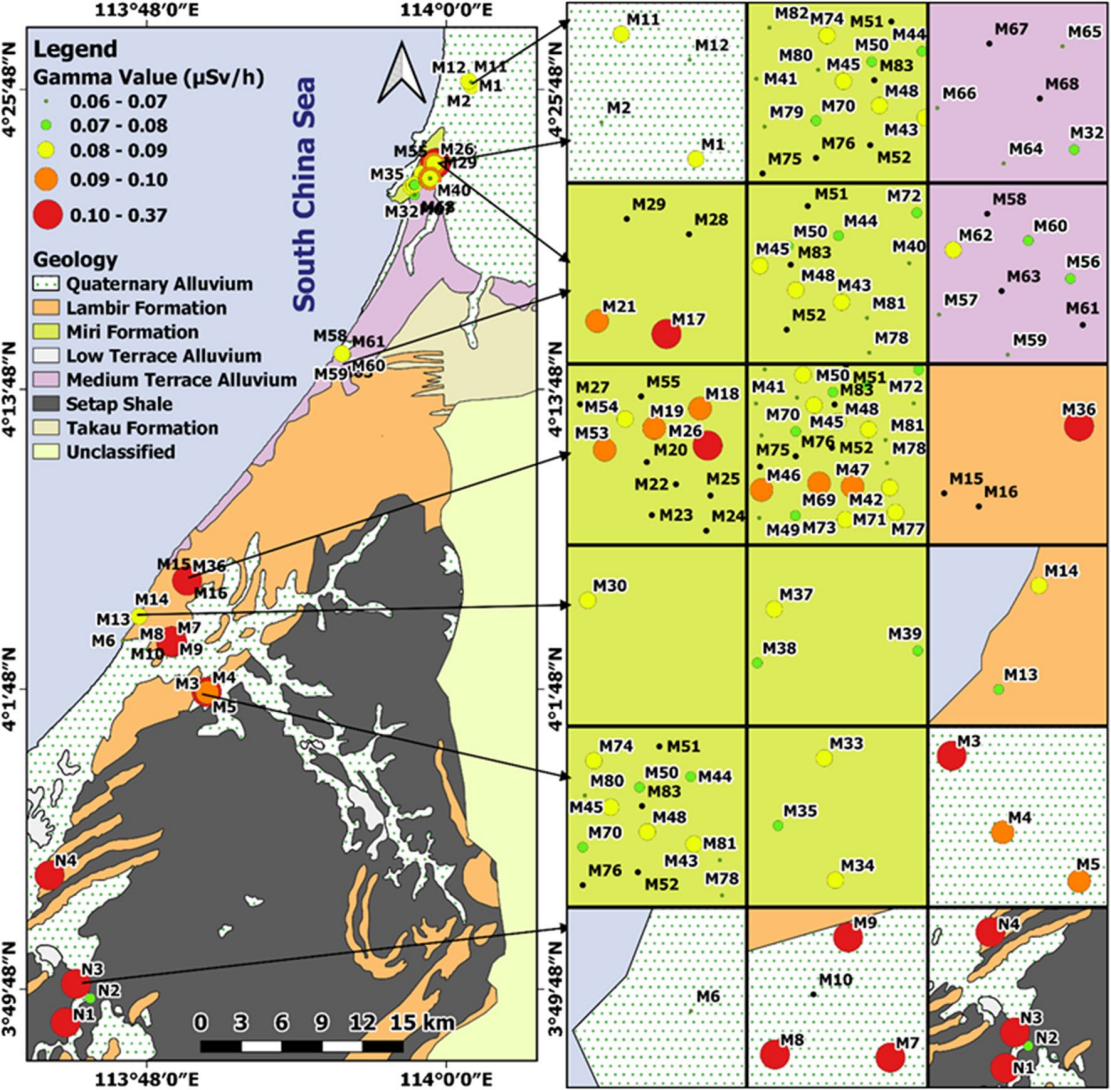
Gamma radiation values at measurement sites of Miri area with geology

The gamma dose rate value represents the overall radioactivity in the environment at 1 m above ground and is a significant parameter for health considerations. On the other hand, beta flux radioactivity is measured directly on the rock surface, closely related to rock type, and is not significant for health considerations. The gamma dose rate map for the wider Miri area is shown in Fig. 9. Miri City in the north with a denser population is underlain mainly by sandstones of the Miri Formation (Hutchison 2005) and does not show high gamma values. Higher gamma values (0.1–0.37 µSv/h) are mainly concentrated around the Bekenu and Niah areas in the south, underlain by rocks of Lambir and Setap Shale formations. These formations have high clay content which results in higher overall radioactivity (Fig. 9).

**Fig. 10:**
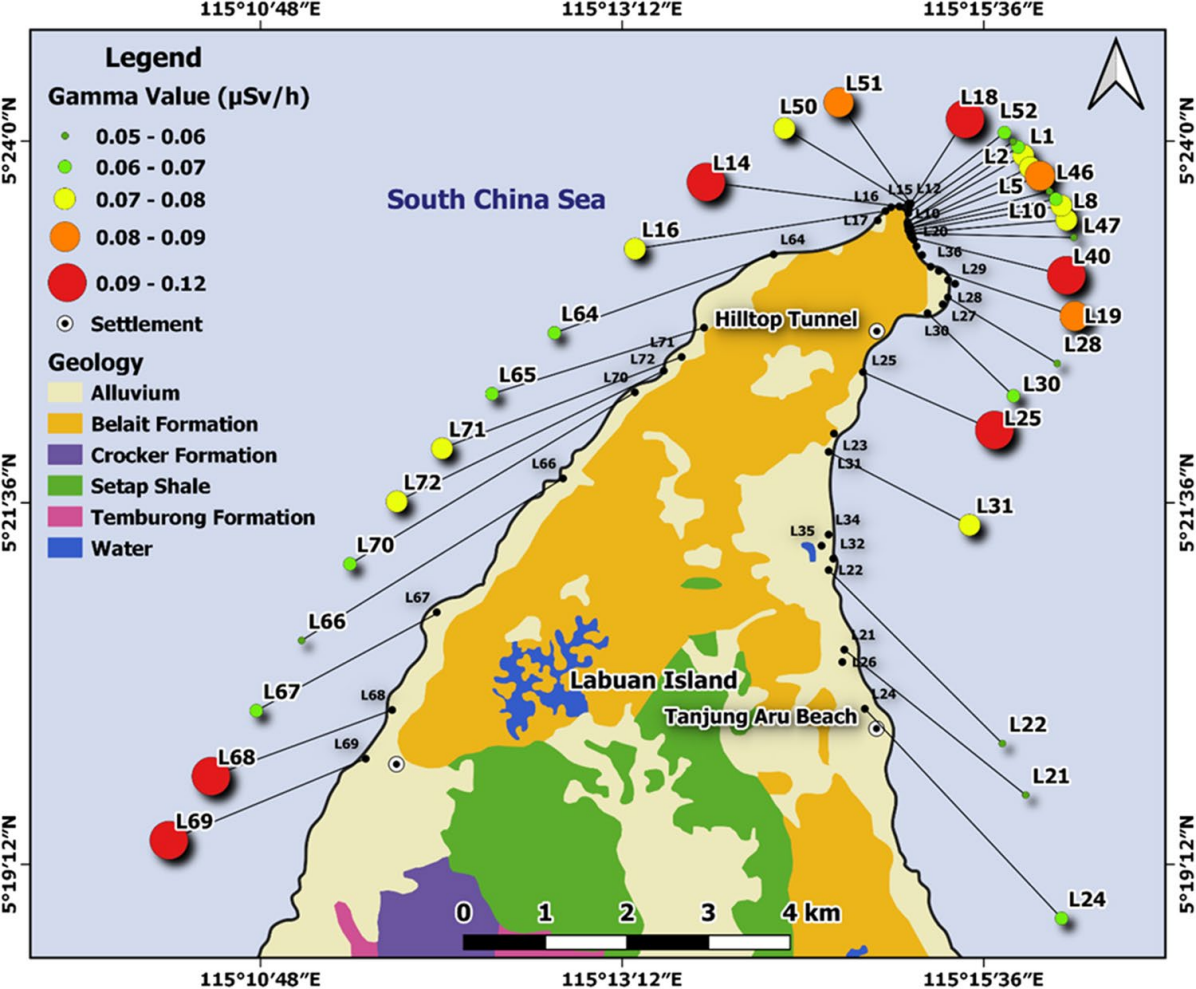
Gamma radiation values at measurement sites of Labuan area with geology

The gamma dose rate map for the North of Labuan is shown in Fig. 10, and it illustrates some variability. Labuan island is mainly underlain by the Belait Formation, which consists of conglomerates, sandstones and shales (Madon 1994), and this area shows gamma dose rate values similar to those observed in Miri. However, mudstones with higher clay contents are concentrated at the northern tip of Labuan island, and this may explain the variability observed, with relatively higher values in that area.

**Fig. 11:**
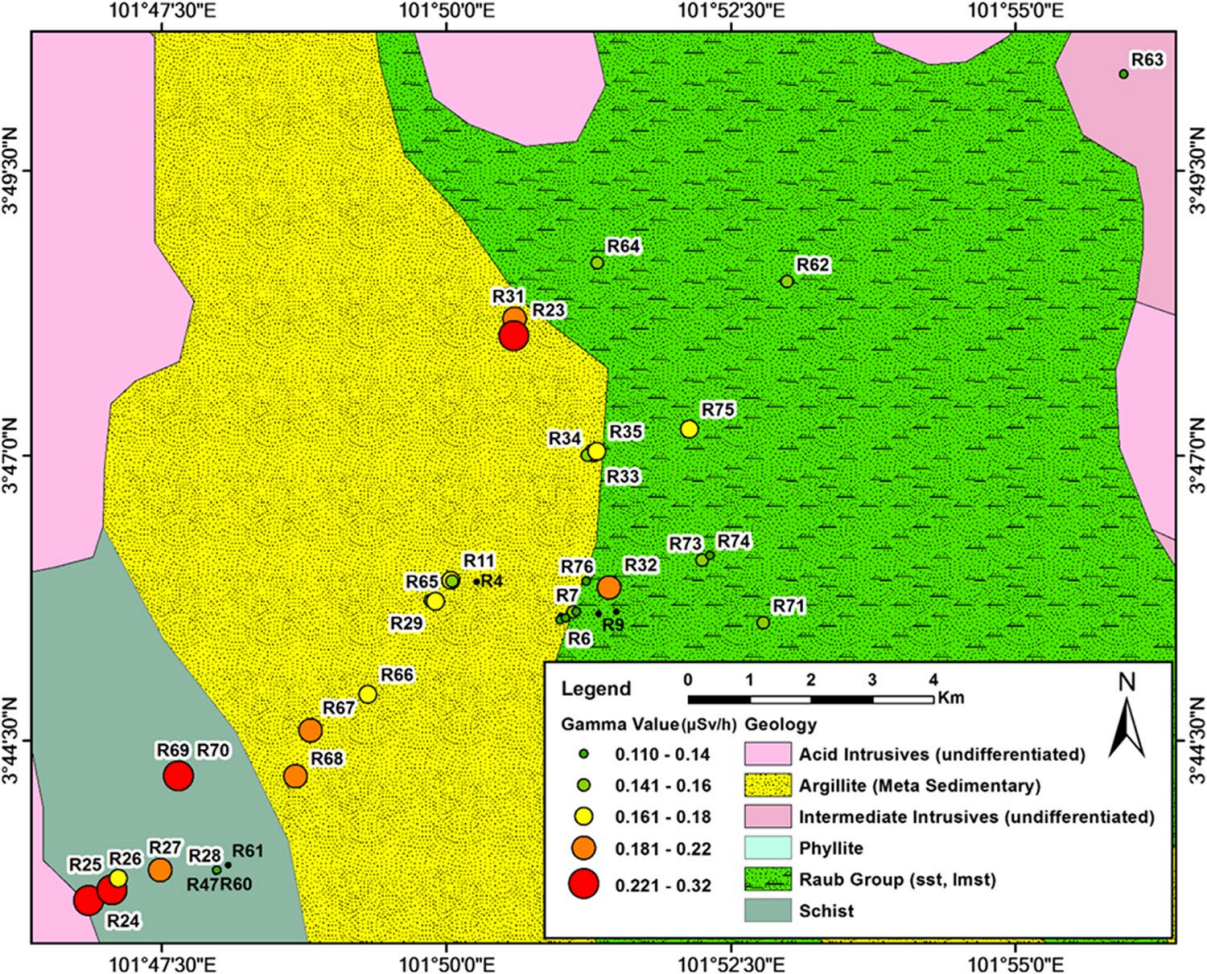
Gamma radiation values at measurement sites of Raub area with geology

The gamma dose map of Raub in Fig. 11 shows significantly higher natural radioactivity in the metamorphic/igneous rock zone, whereas the sedimentary rock zone shows lower natural radioactivity. Rocks like syenite, schist and argillite have higher radioactivity than non-metamorphosed sedimentary rocks.

Ahmad et al. (2014) reported that Malaysia’s maximum TGDR is 1560 nGy/h, and the minimum is 9 nGy/h located in Bukit Merah (Perak) and Pontian (Johor), respectively. The average for Malaysia is 92nGy/h (UNSCEAR 2000). The average values obtained in this study of Labuan, Raub and Miri are 81.1, 175.7 and 91.4 nGy/h, respectively. This study indicates that the Raub area has higher than Malaysian average TGDR while Miri and Labuan fall slightly lower than average. However, the Miri area has higher than the previously studied values (80 and 89 nGy/h) at Curtin University campus and Miri City (Dodge-Wan and Viswanathan 2021; Dodge-Wan et al. 2023). All three sites have higher mean TGRD than the world outdoor average (59 nGy/h) (UNSCEAR 2000).

### Radiation-dependent health risk assessment

Annual effective dose (AED) is a dosimetry unit used by the International Commission of Radiological Protection to calculate human exposure over a year for comparison with safe levels and recommended limits (Sanusi et al. 2016). AED is the sum of both the indoor and outdoor effective dose humans receive in a year (Abu Bakar et al. 2017). For this study, evaluation was carried out using the annual effective dose, based on TGRD rates in nGy/h. The conversion of µSv/h to nGy/h is done by multiplying by the 1000 conversion factor (Dodge-Wan and Viswanathan 2021). The calculation of AED is given below (UNSCEAR 2000):

$$\text{AED}=\left(\text{outdoor TGRD}\times 0.2+\text{indoor TGRD}\times 0.8\right)\times 8760(1\text{ y in hours})\times 0.7\times {10}^{-6}$$

This study has measured only outdoor values of the TGRD. Therefore, site-specific local indoor values cannot be applied due to a lack of data. The outdoor component of the average annual effective dose (AED) for the study areas is shown below:

Labuan

$$\text{outdoor component of AED}=\left(81.1 \times 0.2\right)\times 8760\times 0.7\times {10}^{-6}=0.099 \text{mSv}$$

Raub

$$\text{outdoor component of AED}=\left(175.7 \times 0.2\right)\times 8760\times 0.7\times {10}^{-6}=0.215 \text{mSv}$$

Miri

$$\text{outdoor component of AED}=\left(91.5 \times 0.2\right)\times 8760\times 0.7\times {10}^{-6}=0.112 \text{mSv}$$

For the calculation of AED, both indoor and outdoor values are required. Since site-specific indoor values were not obtained in this study, three different scenarios have been applied to calculate AED as shown in Table 1. These are the following:

1. Use same value for indoor as outdoor average for each area, with the outdoor average being site specific.
2. Use the world indoor average and the site-specific outdoor average.
3. Use the Malaysian indoor average and the site-specific outdoor average.

**Table 1:** Annual effective dose and excess lifetime cancer risk calculated at the three study sites using three different scenarios (1, 2 and 3) for the indoor component

Location	Annual effective dose (in mSv)			Excess lifetime cancer risk (per million people)		
	Scenario 1. Indoor TGRD average is the same as the outdoor average	Scenario 2. World indoor TGRD average	Scenario 3. Malaysia’s indoor TGRD average	Scenario 1. Indoor TGRD average is the same as the outdoor average	Scenario 2. World indoor TGRD average	Scenario 3. Malaysia’s indoor TGRD average
Labuan	0.50	0.51	0.57	1.75 × 10^−3^	1.79 × 10^−3^	2.00 × 10^−3^
Miri	0.56	0.52	0.58	1.96 × 10^−3^	1.82 × 10^−3^	2.03 × 10^−3^
Raub	1.08	0.63	0.69	3.78 × 10^−3^	2.21 × 10^−3^	2.42 × 10^−3^

World indoor and outdoor TGRD average = 84 and 59 nGy/h; Malaysia indoor and outdoor TGRD average = 96 and 92 nGy/h

UNSCEAR (2000) reported that the total and outdoor average worldwide AED are 0.48 and 0.07 mSv, respectively. Outdoor components of AED of study areas except Labuan are significantly higher than worldwide ranging from 0.145 to 0.042 mSv.

The calculated AEDs (Table 1) for the three areas and three scenarios range from 0.50 to 1.08 mSv. The calculated AED values are all below 1 mSv, the dose limit for public exposure suggested by ICRP (1991) except for one calculated value at Raub. The results indicate that overall Miri and Labuan are considered safe for the public as the AED of these areas falls under the standard suggested by ICRP (1991). However, the Raub area, where this study was based on 35 gamma dose readings, indicates a significantly higher outdoor component of AED. It is not known if the indoor gamma dose readings in this area would indicate some shielding, but if buildings do not provide shielding, then the AED may be close to or exceed the standard suggested by ICRP (1991), with higher risk to the public.

Excess lifetime cancer risk (ELCR) quantifies the fatal cancer risk caused by the AED in a human lifetime. It is based on AED and calculated using the equation below (Kolo et al. 2015):

$$\text{ELCR}=\text{ AED }\times \text{DL}\times \text{RF}$$

where AED is the annual effective dose in mSv, DL is the duration of life (70 years) and RF is the risk factor of fatal cancer (0.05 Sv^−1^).

Based only on the outdoor component of AED, rather than total AED, excess lifetime cancer risks for each study area would be:

Labuan

$$\text{outdoor ELCR}=0.099\times {10}^{-3}\times 70\times 0.05=3.45\times {10}^{-4}$$

Raub

$$\text{outdoor ELCR}=0.215\times {10}^{-3}\times 70\times 0.05=7.53\times {10}^{-4}$$

Miri

$$\text{outdoor ELCR}=0.112\times {10}^{-3}\times 70\times 0.05=3.92\times {10}^{-4}$$

However, these values ignore indoor exposure which is applied for 80% of the time to an individual. Hence, in order to provide a better calculation of ELCR, we have used the same three scenarios discussed above to obtain the indoor component of AED, as shown in Table 1. The results indicate that these study areas do not have a high cancer risk to the population as the ELCR values do not exceed the risk level suggested by ICRP (1991), which is 4 × 10^−3^ for the public. Most of the ELCRs obtained (irrespective of the scenarios) are close to or below half that value. However, the ELCR values in all three study areas are higher than the global average ELCR of 1.35 × 10^−3^ (UNSCEAR 2000).

 Labuan and Miri area, predominantly sedimentary rock, show distinctly lower mean TGRD, AED and ELCR than the Raub area, which has igneous, metamorphic rocks that are considered rock types with higher radiation. Overall, natural radioactivity in Labuan and Miri is below the recommended limits indicating no specific radiological health and safety concern for the population. In contrast, the AED in the Raub area is above or close to the recommended limit for public exposure. Further investigation in the Raub area, including indoor gamma dose rates, is suggested to make a more precise estimate of the radiological health risks.

## Conclusion

This research identified 35 different rock types in four diverse geological settings in Peninsular Malaysia (Raub) and East Malaysia (Labuan, Miri, Kundasang). Across these areas, the gamma dose rate varies from 0.05 to 0.37 µSv/h with an average of 0.11 µSv/h. The beta flux ranges from 0.14 to 1.84 CPS with an average of 0.5 CPS. Igneous and metamorphic rock groups have higher natural radioactivity due to the significant quantity of radioactive minerals and radionuclides in the rocks, resulting in higher values of gamma dose rate and beta flux. The lowest gamma dose rate and beta flux values recorded were mainly in mafic igneous rock, sediments, marble and sandstone. Accessory minerals such as zircon, apatite, allanite and titanite and potassium minerals play a crucial role in contributing natural occurring radionuclides (uranium, thorium and potassium) which are responsible for natural radioactivity within the rocks. The spatial variation of gamma dose rate indicates local and regional variations. Higher radiation was observed in the igneous-metamorphic rock zone (Raub) compared to the sedimentary-dominated region (Miri and Labuan).

The annual effective dose for Labuan, Raub and Miri areas is found to be higher than the average worldwide. The calculated excess lifetime cancer risk (ELCR) for Labuan, Miri and Raub is all below the recommended limit suggesting low risk, although they are higher than the global average ELCR. Among the three study areas, Raub contains rock types with higher radioactivity (igneous and metamorphic rock), and the ELCR is closer to the recommended limit than it is in Miri and Labuan areas, with predominantly sedimentary rocks. Overall, the outcome of this research has enhanced the knowledge of natural radioactivity in diverse geological settings in Malaysia, which helps the policy-makers for future development in these regions. It is suggested that further surveys be conducted in the Raub region including indoor TGRD surveys.

### Supplementary Information

Below is the link to the electronic supplementary material.

Supplementary file1 (DOCX 3806 KB)

## Data Availability

The datasets used and analysed during the current study are available from the corresponding author on reasonable request.
