# Supplementary material for: Distribution of natural radioactivity in different geological formations and their environmental risk assessment in Malaysia
Source: Environ Sci Pollut Res Int. 2024 Jun 20;31(30):43292–308. doi: 10.1007/s11356-024-33906-6 (PMC11222256; doi:10.1007/s11356-024-33906-6)
Supplement: Supplementary file 1 — Supplementary file1 (DOCX 3806 KB) [file 11356_2024_33906_MOESM1_ESM.docx]

Supplementary Table 1: Past studies of natural radioactivity worldwide.

| Author | | Study area | | Methods/instruments | | Sample type | | Key findings |
| --- | --- | --- | --- | --- | --- | --- | --- | --- |
| (Rani & Singh, 2005) | | Himachal Pradesh, India | | Gamma spectrometry | | Soil | | Assessed and measured the natural radioactivity in the study area |
| (Xu et al., 2006) | | N/A | | N/A | | Cordierites | | Assessed the radiation properties of substituted cordierites via structure, chemical |
| (Chiozzi et al., 2007) | | Lipari volcanic island, Italy | | Gamma spectrometry | | Geological formation | | Radiometric survey on hydrothermal alteration area |
| (Agbalagba & Onoja, 2011) | | Biseni, Nigeria | | Gamma spectrometry | | Soil, Sediment, water | | Assessed and measured the natural radioactivity in the study area |
| (Cetin et al., 2012) | | Turkey | | Gamma spectrometry | | Granite | | Measured the natural radioactivity in the area and analysed mineralogically |
| (Korkulu & Özkan, 2013) | | Kocaeli, Turkey | | Gamma spectrometry | | Beach sand | | Measured the natural radioactivity and gamma-ray spectrum in the area |
| (Uyanik et al., 2013) | Isparta, Turkey | | NaI survey meter | | Soil | | Analysed the earthquake risk and radiological properties of the study area | |
| (Garcia-Orellana et al., 2013) | Peniscola, Spain | | Gamma spectrometry | | Water, soil | | Investigated the submarine groundwater discharge and the natural radioactivity accumulation | |
| (Manjunatha et al., 2013) | | Chikmagalur, India | | Gamma spectrometry | | Geological formation | | Compared different rock formations with the distribution of the radionuclide |
| (Szabó et al., 2013) | | Hungarian | | Gamma spectrometry | | Building materials | | Compared radioactivity of natural and artificial building materials |
| (Ramasamy et al., 2014) | | Tamilnadu, India | | Gamma spectrometry | | River sediments | | Analysed mineralogical role in natural radioactivity using the FTIR technique |
| (Durašević et al., 2014) | | Serbia | | Gamma spectrometry | | Lignite | | Analysed and assessed the natural radioactivity of lignite samples from open pit mines |
| (Saleh & Abu Shayeb, 2014) | | Jordan | | Alpha and Beta spectrometry | | Soil | | Assessed and measured the natural radioactivity in the study area |
| (Chandrasekaran et al., 2015) | | Tamilnadu, India | | Gamma spectrometry | | Soil | | Analysed mineralogical role in natural radioactivity using the FTIR technique |
| (Alshamsia et al., 2015) | | United Arab Emirates (UAE) | | ICP- MS | | Carbonate rock | | Evaluated the natural radioactivity of carbonate as a building material |
| (Cinelli & Tondeur, 2015) | | Walloon region, Belgium | | N/A | | N/A | | Reviewed and examined the indoor radon data using a log-normal trend |
| (Al-Ghamdi et al., 2016) | | Saudi Arabian gulf | | Gamma spectrometry | | Sediment, seawater | | Assessed and measured the natural radioactivity in the study area |
| (Guagliardi et al., 2016) | | Calabria, Italy | | Gamma spectrometry | | Geological formation, soil | | Assessed the effects of source rock, and climate on natural radioactivity |
| (Punniyakotti & Ponnusamy, 2017) | | Tamilnadu coast, India | | Gamma spectrometry | | Intertidal sand | | Analysed mineralogical role in natural radioactivity using FTIR and XRD technique |
| (Marcon et al., 2017) | | Lucrécia city, Brazil | | Handheld Gamma-Ray Spectrometer RS-125 Super-SPEC | | water | | Analysed the mutagenic potential of natural radioactivity in the aspect of metal, cyanobacteria and total radiation |
| (Korkmaz et al., 2017) | | Karadag mountain, Turkey | | Gamma spectrometry | | Soil | | Assessed and measured the natural radioactivity in the study area |
| (Shohda et al., 2018) | | Egypt | | Gamma spectrometry | | Marble, Granite, Serpentine | | Radiometric and mineralogy analysis for Egyptian ornamental stones |
| (Sankaran Pillai et al., 2018) | | Tamilnadu, India | | N/A | | N/A | | Reviewed the variation of natural radioactivity of Tamil Nadu |
| (Dżaluk et al., 2018) | Opava mountain, Poland | | Gamma spectrometry | | Igneous rock | | Compared the laboratory and in situ natural radioactivity measurements | |
| (Ribeiro et al., 2018) | Rio de Janeiro, Brazil | | Gamma spectrometry | | Geological formation, soil | | Investigated the radiological characterisation of geological units | |
| (El Zrelli et al., 2019) | | Tunisia | | Gamma spectrometry | | Phosphate rock, industrial waste | | Assessed radiation hazards of the coastal phosphate treatment plant |
| (Silva et al., 2019) | | Paraiba do Sul, Brazil | | ICP-MS | | Mineral water | | Determined the natural radioactivity in the mineral water |
| (Alajeeli et al., 2019) | | Libya | | Gamma spectrometry | | Soil | | Assessed the radiological hazard of the agriculture project |
| (Cinelli et al., 2019) | | Europe | | N/A | | Geological formation, soil | | Concluded and reviewed various natural radiation maps |
| (Ashrafi & Jahanbakhsh, 2019) | | Azerbaijan, Iran | | Gamma spectrometry | | Granite | | Measured the natural radioactivity using beta-gamma coincidence and maximum likelihood |
| (Missimer et al., 2019) | | Florida, USA | |  | | Geological formation, soil, water | | Summarised and reviewed natural radiation in the study area |
| (Dicu et al., 2019) | | Bihor County, Romania | | RaThoGamma Kit | | Water | | Assessed the annual effective dose from natural radioactivity sources |
| (Kazumasa et al., 2020) | | Vietnam | | Gamma spectrometry | | Soil | | Examined the gamma distribution in all of Vietnam |
| (Pourimani et al., 2020) | | Arak, Iram | | Gamma spectrometry | | Sediment and water | | Assessed and measured the natural radioactivity in the study area |
| (Arneodo et al., 2020) | | Arabian Peninsula and gulf | | Gamma spectrometry | | Carbonate Formation | | Evaluated the uranium content of carbonate in petroleum reservoirs |
| (Mantero et al., 2020) | | Sweden | | Gamma and alpha spectrometry | | Water, sediments | | Assessed the natural radioactivity and elementary characterisation of the study area |
| (Kaya et al., 2020) | | Gumushane, Turkey | | Gamma spectrometry | | Water, soil | | Measured and mapped the natural background radioactivity of the study area |
| (Szkliniarz et al., 2021) | Polkowice-Sieroszowice mine, Poland | | Gamma spectrometry | | Geological formation | | Assessed the characteristics of natural background radiation in underground | |
| (Malczewski et al., 2021) | Jeronym mine, Czech Republic | | Gamma spectrometry | | Geological formation | | Measured the natural radioactivity of rocks in the mine | |
| (Manisa et al., 2021) | Corlu, Turkey | | NaI survey meter and Gamma spectrometry | | Soil, water | | Assessed and measured the natural radioactivity in the study area | |
| (Özden & Aközcan, 2021) | Aliaga Bay, Turkey | | Gamma spectrometry | | Sediments | | Assessed and measured the natural radioactivity and radiological hazards in the study area | |
| (Ismail et al., 2021) | Iraqi Kurdistan | | Gamma spectrometry | | Beach sands | | Measured the natural radioactivity in the study area | |
| (Melgar & García, 2021) | Galicia, Spain | | Gamma spectrometry | | Soil and Mushrooms | | Examined the K-40 level in soil and mushrooms | |
| (Kapanadze et al., 2021) | Imereti, Georgia | | Gamma spectrometry | | Soil | | Assessed and measured the natural radioactivity in the study area | |
| (Dinis et al., 2021) | Portugal | | Gamma spectrometry | | Soil and air | | Assessed and measured the natural radioactivity around the coal-fired plant | |
| (Vásconez-Maza et al., 2022) | Campo de Cartagena, Spain | | Portable Geiger Radex RD1503 | | Phosphogypsum deposits | | Examined the radiological, geophysical, and geochemical features of Phosphogypsum deposits | |
| (Amaral et al., 2022) | Recife, Brazil | |  | | Water | | Conducted a risk survey of radium in groundwater for the population of the study area | |
| (Younis et al., 2022) | Himalayas, Pakistan | | Gamma spectrometry | | Granitoids | | Assessed and measured the gamma radioactivity and radiation risk of specific rocks in the study area | |
| (Aruta et al., 2022) | Yerevan, Armenia | | Gamma spectrometry | | Soil | | Analysed the distribution patterns of radionuclides activities using Multifractal Inverse Distance Weighting | |
| (Nursapina et al., 2022) | Kazakhstan | | Alpha spectrometry | | Soil | | Assessed the effect of mineral fertilisers in the soil on radish | |
| (Edomskaya et al., 2022) | Eastern Europe | | Gamma and alpha spectrometry | | Soil | | Estimated the global fallout of radionuclides in the study area | |
| (Xu et al., 2022) | Xuancheng, China | | Gamma spectrometry | | Geological formation, soil | | Assessed the natural radioactivity using airborne gamma-ray spectrometric view | |
| (Salahel din, 2022) | Abu Simbel, Egypt | | Gamma spectrometry | | Soil | | Assessed and measured the natural radioactivity in the study area | |
| (Yakovlev et al., 2022) | Kola Peninsula, Russia | | Alpha spectrometry | | Surface water | | Health assessment of surface water of rivers and lakes in the study area | |
| (Nguyen & Trinh, 2022) | Nghe An, Vietnam | | Gamma spectrometry | | Soil | | Measured the natural radioactivity of soil in monazite placer | |
| (Gibaga et al., 2022) | Philippines | | Gamma spectrometry | | Mine waste | | Conducted radiological risk assessment in mines | |

Supplementary Table 2. Past studies of natural radioactivity in Malaysia.

| Author | Study area | Methods/instruments | Sample type | Key findings |
| --- | --- | --- | --- | --- |
| (Ramli et al., 2003) | Kota Tinggi, Johor | NaI survey meter | Geological formation, soil | Constructed statistical prediction of gamma dose rate |
| (Ramli et al., 2005) | Melaka | NaI survey meter | Soil | Assessed the environmental radiological health |
| (Gillmore et al., 2005) | Niah cave, Sarawak | Alpha track radon detector | Soil, Limestone | Assessed the potential risk of Radon in the cave |
| (Abdul Rahman & Ramli, 2007) | Ulu Tiram, Johor | NaI survey meter | Soil | Measured the radioactivity level of ^238^U and ^232^Th |
| (Lee et al., 2009) | Kinta, Perak | NaI survey meter and Gamma spectrometry (HPGe) | Soil | Monitored and measured the dose rate and activity concentration |
| (Yii & Wan Mahmood, 2011) | East coast, west Malaysia | Gamma spectrometry (HPGe) and alpha spectrometry (PIPS) | Sediment, Biota | Measured the radioactivity of ^137^Cs (plutonium isotopes) |
| (Wan Mahmood & Yii, 2012) | East coast, west Malaysia | Gamma spectrometry (HPGe) | Sediment core | Measured the marine radioactivity of ^228^Ra, ^226^Ra |
| (Almayahi et al., 2012) | Penang | NaI survey meter and Gamma spectrometry (HPGe) | Soil | Assessed the radiological hazards from the activity concentration of ^238^U, ^226^Ra, ^40^K and ^232^Th |
| (Saleh et al., 2013) | Segamat, Johor | NaI survey meter | Geological formation, soil | Assessed the ^226^Ra, ^40^K and ^232^Th concentration |
| (Gabdo et al., 2016) | Pahang | NaI survey meter | Soil | Measured the terrestrial gamma dose rate |
| (Saleh et al., 2014) | Kluang, Johor | Gamma spectrometry (HPGe) | Soil | Assessed the health hazards |
| (Sanusi et al., 2014) | Selangor, Kuala Lumpur, Putrajaya | NaI survey meter | Geological formation, soil | Produced isodose map of gamma dose rate of the study areas |
| (Alnour et al., 2014) | Johor | Gamma spectrometry (HPGe) | Geological formation | Measured the natural radioactivity levels in rocks |
| (Kolo et al., 2015) | Kuantan, Pahang | Gamma spectrometry (HPGe) | Soil | Evaluated the radiological risks around Lynas Advanced Material Plant |
| (Saleh et al., 2015) | Johor | NaI survey meter | Geological formation, soil | Constructed a terrestrial gamma dose rate prediction based on geological units |
| (Gabdo et al., 2016) | Pahang | Gamma spectrometry (HPGe) | Geological formation, soil | Measured the natural radioactivity |
| (Garba et al., 2016) | Terengganu | NaI survey meter | Soil | Assessed the potential chronic exposure to low-dose natural radioactivity |
| (Alzubaidi et al., 2016) | Kedah | Gamma spectrometry (HPGe) | Soil | Assessed the natural radioactivity of agricultural and virgin soil |
| (Sanusi et al., 2016) | Western peninsular Malaysia | NaI survey meter | Geological formation, soil | Investigated the influence of geological units on natural gamma radiation exposure |
| (Abu Bakar et al., 2017) | Fraser's Hill, Pahang | Gamma spectrometry (HPGe) | Soil | Measured the natural radioactivity |
| (Shuaibu et al., 2017) | Penang | Gamma spectrometry (HPGe) | Sand | Assessed the natural radioactivity and gamma-ray dose in monazite-rich sand |
| (Izham et al., 2017) | West Sarawak | NaI survey meter | Soil | Measured and provided an overview of the gamma dose rate in west Sarawak |
| (Garba et al., 2019) | Kelantan | Gamma spectrometry (HPGe) | Soil | Measured and assessed the natural radioactivity and radiation hazards |

Supplementary Table 3. Mineralogy of collected samples in the study areas. Highest (pale yellow), lowest (green)

Supplementary Figure 1: Thin section photos of selected samples 1, 2: Zircon and cordierite with haloes, PPL and XPL(Monzogranite) 3: Allanite in chlorite, PPL (Monzogranite), 4: Biotite, muscovite and K-feldspar in Monzogranite, PPL, 5, 6: Zircon inclusion in biotite, PPL, XPL (Monzogranite), 7, 8: Allanite in argillite, PPL, XPL, 9: Zircon in metasandstone, XPL, 10: Apatite, epidote, allanite and K-feldspar in syenite, PPL, 11, 12: Allanite and epidote in syenite, PPL, XPL, 13, 14: Allanite in hornblendes (Syenite), PPL, XPL, 15, 16: pyrite and muscovite in pyrite-graphite-quartz schist, PPL, XPL, 17, 18: Glauconite in litharenite, PPL, XPL, 19: Biotite in sublitharenite, PPL, 20: sublitharenite, XPL.


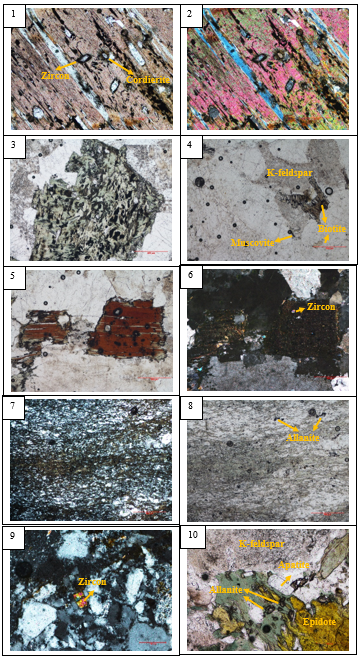

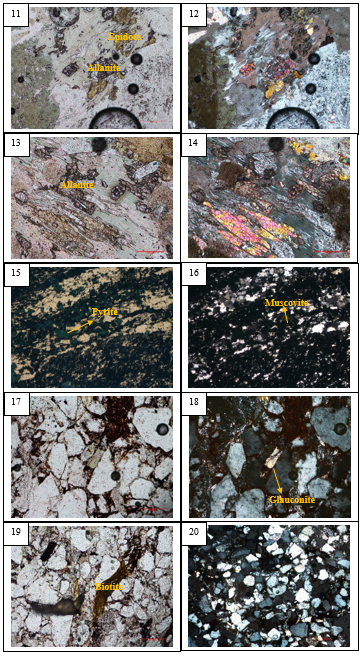

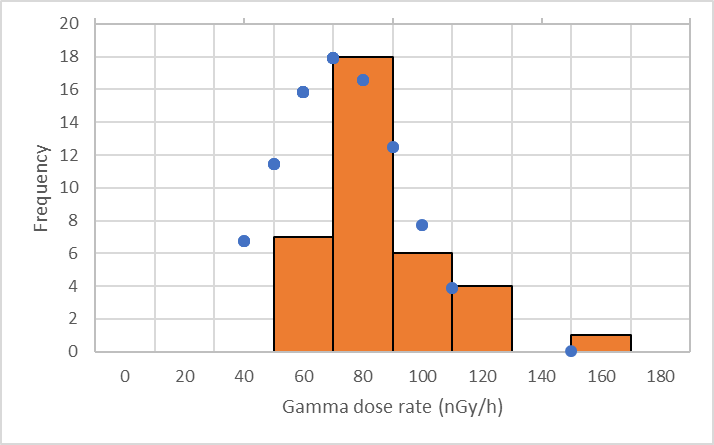


a


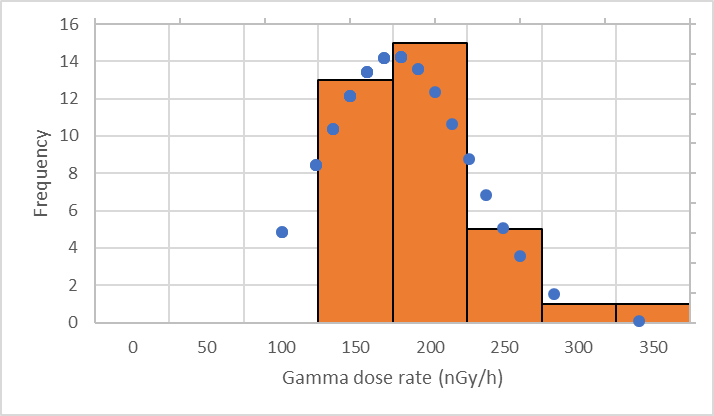


b


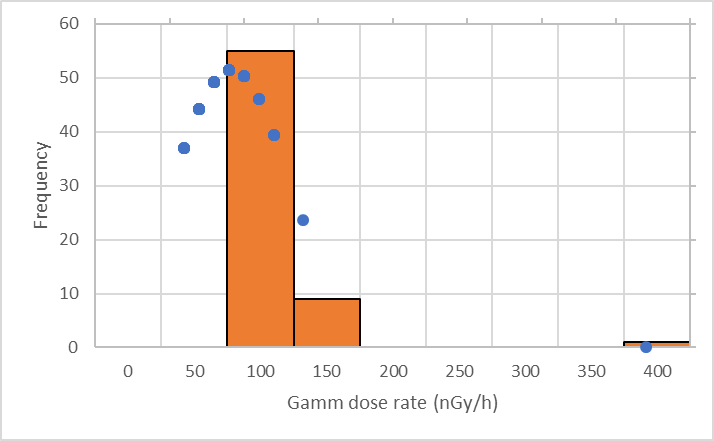


c

Supplementary Figure 2. Frequency distribution histogram of gamma dose rate a: Labuan, b: Raub, c: Miri.


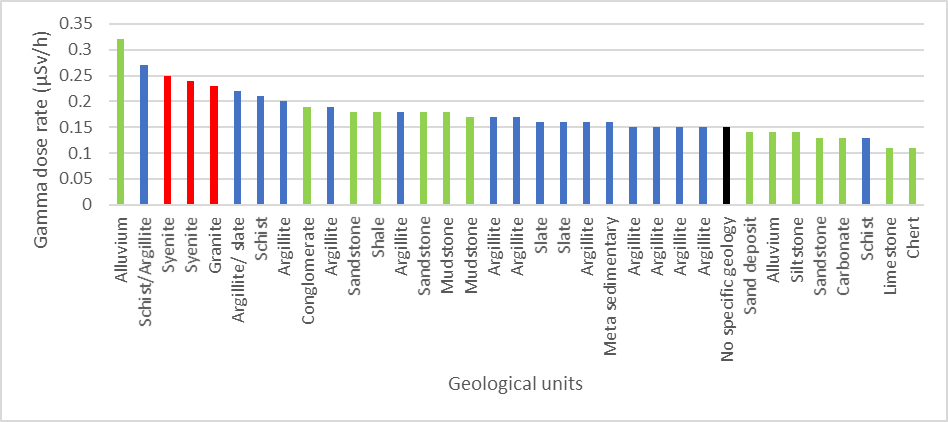


a


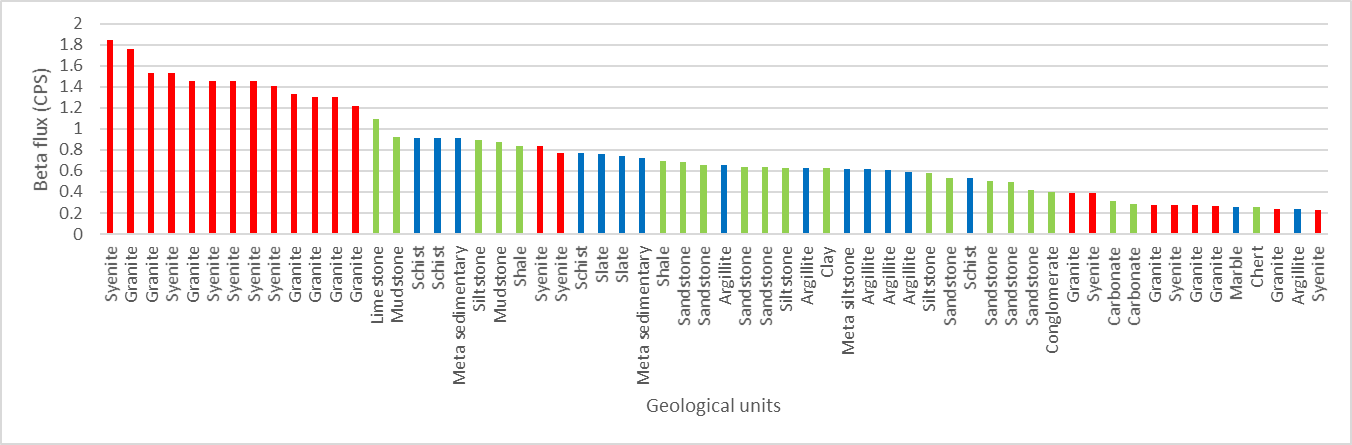


b

Supplementary Figure 3. Bar chart of natural radioactivity data in the Raub area. a: Gamma dose rate, b: Beta flux. Igneous rock (red), metamorphic rock (blue), sediment and sedimentary rock (green)


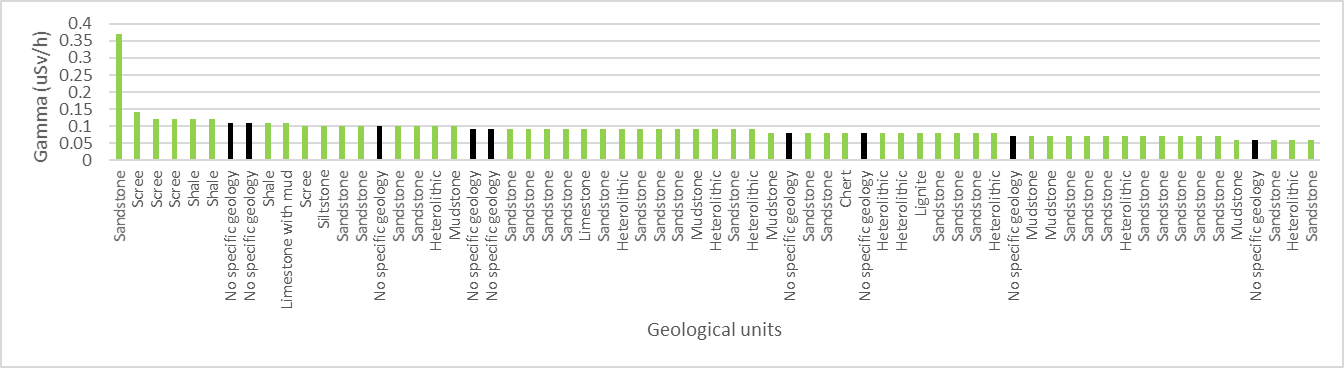


a


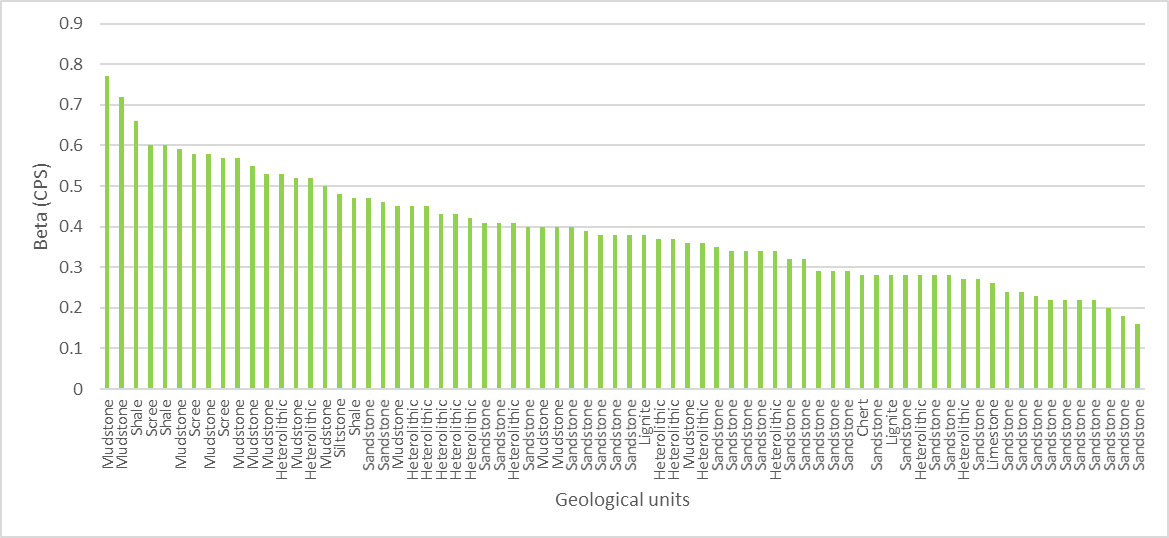


b

Figure 8. Bar chart of natural radioactivity data in the Miri area. a: Gamma dose rate, b: Beta flux.

Supplementary Figure 4. Bar chart of natural radioactivity data in the Miri area. a: Gamma dose rate, b: Beta flux.


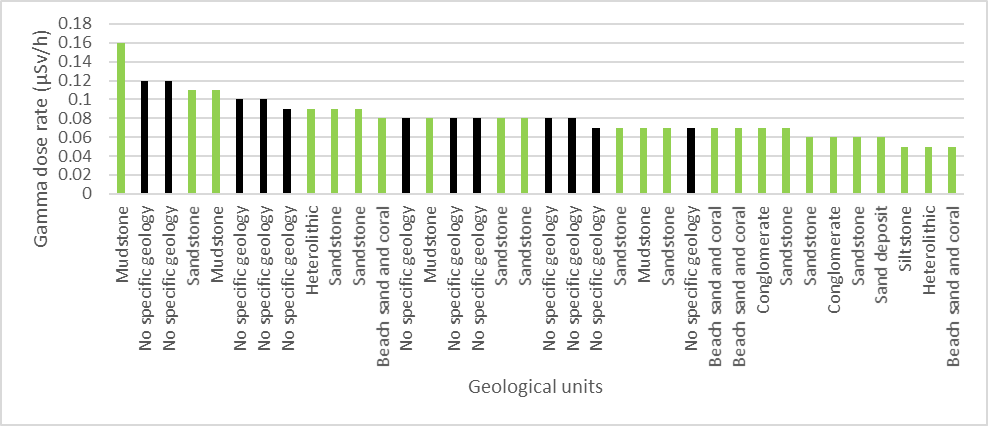


a


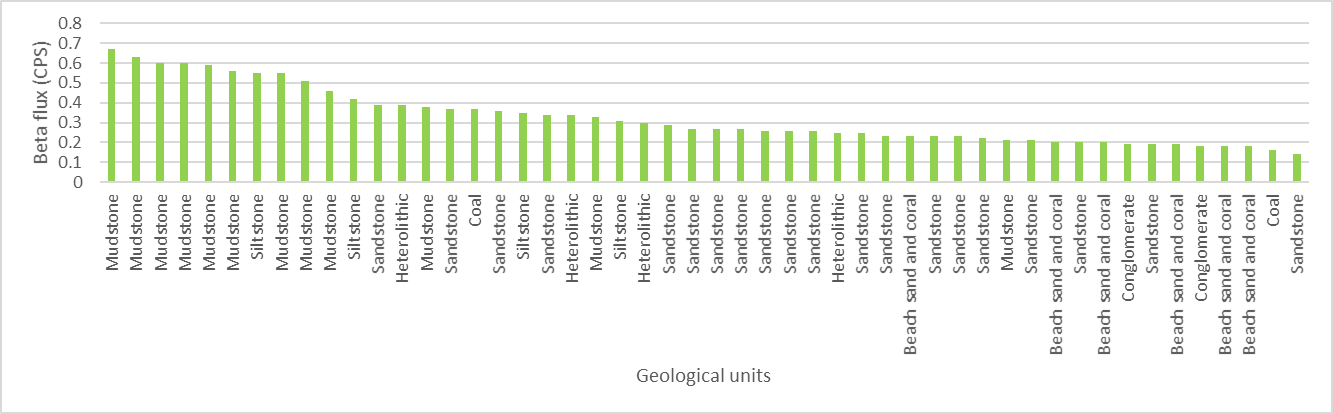


b

Supplementary Figure 5. Bar chart of natural radioactivity data in the Labuan area. a: Gamma dose rate, b: Beta flux.


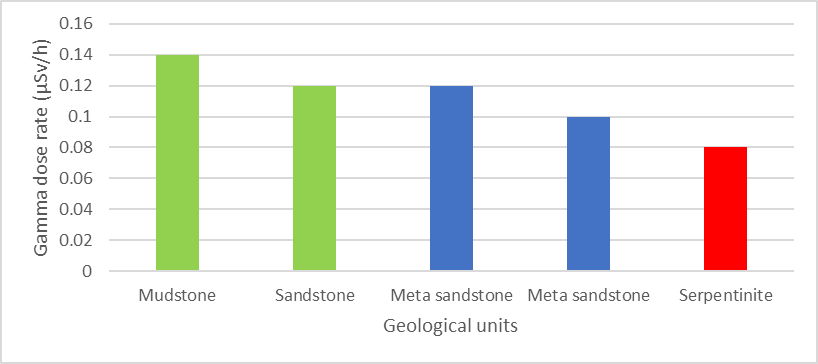


a


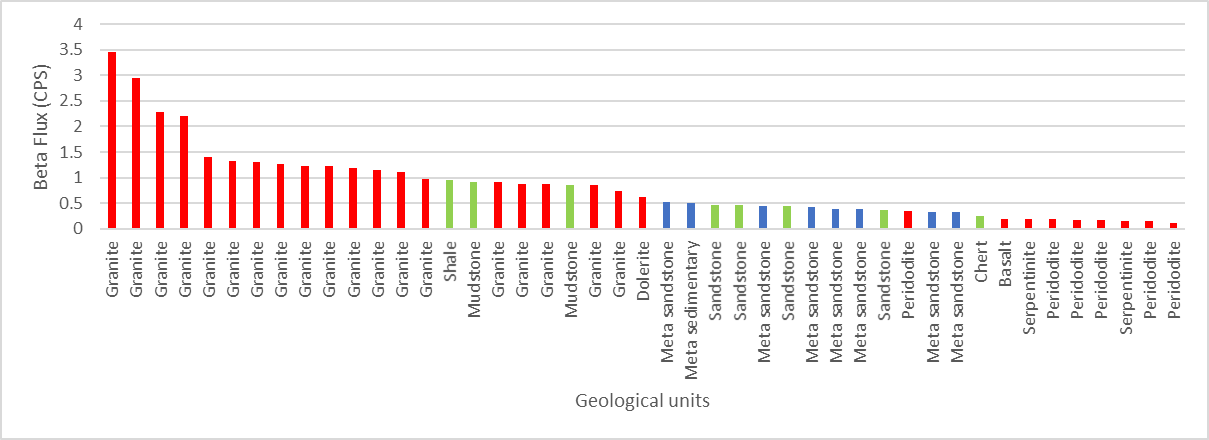


b

Supplementary Figure 6. Bar chart of natural radioactivity data in the Kundasang area

**References:**

Abdul Rahman A. T, Ramli A. T (2007) Radioactivity levels of ^238^U and ^232^Th, the α and β activities and associated dose rates from surface soil in Ulu Tiram, Malaysia. Journal of Radioanalytical and Nuclear Chemistry 273(3): 653–657. https://doi.org/10.1007/s10967-007-0926-2

Agbalagba E. O, Onoja R. A (2011) Evaluation of natural radioactivity in soil, sediment and water samples of Niger Delta (Biseni) flood plain lakes, Nigeria. Journal of Environmental Radioactivity 102(7): 667–671. https://doi.org/10.1016/j.jenvrad.2011.03.002

Alajeeli A, Elmahroug Y, Mohammed S, Trabelsi A (2019) Determination of natural radioactivity and radiological hazards in soil samples: Alhadba and Abuscabh agriculture projects in Libya. Environmental Earth Sciences 78(6): 1–8. https://doi.org/10.1007/s12665-019-8213-4

Al-Ghamdi H, Al-Muqrin A, El-Sharkawy A (2016) Assessment of natural radioactivity and 137Cs in some coastal areas of the Saudi Arabian gulf. Marine Pollution Bulletin 104(1–2): 29–33. https://doi.org/10.1016/j.marpolbul.2016.01.058

Almayahi B. A, Tajuddin A. A, Jaafar M. S (2012) Effect of the natural radioactivity concentrations and 226 Ra / 238 U disequilibrium on cancer diseases in Penang, Malaysia. Radiation Physics and Chemistry 81(10): 1547–1558. https://doi.org/10.1016/j.radphyschem.2012.03.018

Alshamsia D, Murada A, Aldahana, Hou X. L (2015) Natural radioactivity of carbonate rocks as materials for construction and cement industry. ICEG, 271–273. https://doi.org/10.1190/iceg2015-076

Alzubaidi G, Hamid FB, Abdul Rahman I (2016) Assessment of Natural Radioactivity Levels and Radiation Hazards in Agricultural and Virgin Soil in the State of Kedah, North of Malaysia. Scientific World Journal 6178103. doi: 10.1155/2016/6178103.

Amaral R. dos S, Santos Júnior J. A. dos, Fernández Z. H, Paiva Melo N. M. de, Casado da Silva A. N, Marques do Nascimento Santos J, Cavalcanti Freire Bezerra M. B, Lopes de Barros Correia F., Antônio da Silva A, Alves A. V, D’Andrada Bezerra L. R (2022) Risk survey for the population of Recife and neighbouring cities due to the occurrence of radium in groundwater. Journal of Environmental Radioactivity 247. https://doi.org/10.1016/j.jenvrad.2022.106868

Arneodo F, Balata M, Benabderrahmane M. L, Bruno G, Di Giovanni A, Fawwaz O, Laubenstein M, Manenti L, Nisi S, Richter R. B (2020) Characterisation of Naturally Occurring Radioactive Material (NORM) Generated from the Lower Cretaceous Carbonate Formations in the Arabian Peninsula and Gulf. Berg Huettenmaenn Monatsh, 165(8): 353–363. https://doi.org/10.1007/s00501-020-01011-z

Aruta A, Sahakyan L, Tepanosyan G, Movsisyan N, Belyaeva O, Albanese S (2022) Multifractal features of activity concentration and stochastic risk assessment of naturally occurring and technogenic radionuclides in the soil of Yerevan, Armenia. Environmental Pollution 301, 119000. https://doi.org/10.1016/j.envpol.2022.119000

Ashrafi S, Jahanbakhsh O (2019) Measurement of natural radioactivity of Iranian granite samples using beta-gamma coincidence spectrometer and maximum likelihood method. Environmental Earth Sciences 78(15): 1–8. https://doi.org/10.1007/s12665-019-8434-6

Cetin E, Altinsoy N, Örgün Y (2012) Natural radioactivity levels of granites used in Turkey. Radiation Protection Dosimetry 151(2): 299–305. https://doi.org/10.1093/rpd/ncs007

Cinelli G, Tollefsen T, Bossew P, Gruber V, Bogucarskis K, De Felice L, De Cort M (2019) Digital version of the European Atlas of natural radiation. Journal of Environmental Radioactivity 196: 240–252. <https://doi.org/10.1016/j.jenvrad.2018.02.008>

Cinelli G, Tondeur F (2015) Log-normality of indoor radon data in the Walloon region of Belgium. Journal of Environmental Radioactivity 143: 100–109. https://doi.org/10.1016/j.jenvrad.2015.02.014

Dicu T, Burghele B. D, Cucos A, Mishra R, Sapra B. K (2019) Assessment of annual effective dose from exposure to natural radioactivity sources in a case-control study in Bihor county, Romania. Radiation Protection Dosimetry 185(1): 15–24. https://doi.org/10.1093/rpd/ncy211

Dinis M. de L, Fiúza A, Góis J, de Carvalho J. S, Meira Castro A. C (2021) Assessment of natural radioactivity, heavy metals and particulates matter in air and soil around a coal-fired power plant—an integrated approach. Atmosphere 12(1433): 1–20. https://doi.org/10.3390/atmos12111433

Durašević M, Kandić A, Stefanović P, Vukanac I, Šešlak B, Milošević Z, Marković T (2014) Natural radioactivity in lignite samples from open pit mines "Kolubara", Serbia - risk assessment. Applied Radiation and Isotopes 87: 73–76. https://doi.org/10.1016/j.apradiso.2013.11.096

Dżaluk A, Malczewski D, Żaba J, Dziurowicz M (2018) Natural radioactivity in granites and gneisses of the Opava Mountains (Poland): a comparison between laboratory and in situ measurements. Journal of Radioanalytical and Nuclear Chemistry 316 (1): 101–109. https://doi.org/10.1007/s10967-018-5726-3

Edomskaya M. A, Lukashenko S. N, Stupakova G. A, Kharkin P. V, Gluchshenko V. N, Korovin S. V (2022) Estimation of radionuclides global fallout levels in the soils of CIS and eastern Europe territory. Journal of Environmental Radioactivity 247, 106865. https://doi.org/10.1016/j.jenvrad.2022.106865

El Zrelli R, Rabaoui L, van Beek P, Castet S, Souhaut M, Grégoire M, Courjault-Radé P (2019) Natural radioactivity and radiation hazard assessment of industrial wastes from the coastal phosphate treatment plants of Gabes (Tunisia, Southern Mediterranean Sea). Marine Pollution Bulletin 146: 454–461. https://doi.org/10.1016/j.marpolbul.2019.06.075

Gabdo H. T, Ramli A. T, Saleh M. A, Garba,N. N, Sanusi M (2016) Natural radioactivity measurements in Pahang State, Malaysia. Isotopes in Environmental and Health Studies 52(3): 298–308. https://doi.org/10.1080/10256016.2016.1128428

Garba N. N, Ramli A. T, Saleh M. A, Gabdo H. T, Sanusi M. S, Aliyu A. S (2016) The potential health hazards of chronic exposure to low-dose natural radioactivity in Terengganu, Malaysia. Environmental Earth Sciences 75(5): 1–12. https://doi.org/10.1007/s12665-015-5217-6

Garba N. N. Ramli A. T, Saleh M. A, Gabdo H. T (2019) Human and Ecological Risk Assessment: An International Natural radioactivity and associated radiation hazards in soil of Kelantan, Malaysia. Human and Ecological Risk Assessment 25(7): 1707–1717. https://doi.org/10.1080/10807039.2018.1474433

Garcia-Orellana J, Rodellas V, Casacuberta N, Lopez-Castillo E, Vilarrasa M, Moreno V, Garcia-Solsona E, Masqué P (2013) Submarine groundwater discharge: Natural radioactivity accumulation in a wetland ecosystem. Marine Chemistry 156: 61–72. https://doi.org/10.1016/j.marchem.2013.02.004

Gibaga C. R. L, Samaniego J. O, Tanciongco A. M., M, R. N, Montano M. O, Gervasio J. H. C, Reyes R. C. G, Peralta M. J. V (2022) Pollution and radiological risk assessments of mine wastes from selected legacy and active mines in the Philippines Pollution and radiological risk assessments of mine wastes from selected legacy and active mines in the Philippines. Journal of Degraded and Mining Lands Management 9(4): 3621–3633. <https://doi.org/10.15243/jdmlm.2022.094.3621>

Gillmore G, Gilbertson D, Grattan J, Hunt C, McLaren S, Pyatt B, Banda R. M, Barker G, Denman A, Phillips P, Reynolds T (2005) The potential risk from 222radon posed to archaeologists and earth scientists: Reconnaissance study of radon concentrations, excavations, and archaeological shelters in the Great Cave of Niah, Sarawak, Malaysia. Ecotoxicology and Environmental Safety 60(2): 213–227. https://doi.org/10.1016/j.ecoenv.2003.12.014

Guagliardi I, Rovella N, Apollaro C, Bloise A, De Rosa R, Scarciglia F, Buttafuoco G (2016) Effects of source rocks, soil features and climate on natural gamma radioactivity in the Crati valley (Calabria, Southern Italy). Chemosphere 150: 97–108. https://doi.org/10.1016/j.chemosphere.2016.02.011

Ismail A. H, Hussein Z. A, Aladdin D. H (2021) Measurement of Natural Radioactivity in Samples of Beach Sands (Rivers and Lakes) in the Iraqi Kurdistan Region. Radiochemistry 63(3): 389–394. https://doi.org/10.1134/S1066362221030176

Kaya S, Kaya A, Çelik N, Kara R. T, Taşkın H, Koz B (2020) Determination of the environmental natural radioactivity and mapping of natural background radioactivity of the Gumushane province, Turkey. Journal of Radioanalytical and Nuclear Chemistry 326(2): 933–957. https://doi.org/10.1007/s10967-020-07390-4

Korkmaz M. E, Agar O, Uzun E (2017) Assessment of natural radioactivity levels for Karadaǧ Mountain, Turkey. International Journal of Radiation Research 15(4): 399–406. https://doi.org/10.18869/acadpub.ijrr.15.4.399

Korkulu Z, Özkan N (2013) Determination of natural radioactivity levels of beach sand samples in the black sea coast of Kocaeli (Turkey). Radiation Physics and Chemistry 88, 27–31. <https://doi.org/10.1016/j.radphyschem.2013.03.022>

Lee S. K, Wagiran H, Ramli A. T, Apriantoro N. H, Khalik Wood A (2009) Radiological monitoring: terrestrial natural radionuclides in Kinta District, Perak, Malaysia. Journal of Environmental Radioactivity 100(5): 368–374. <https://doi.org/10.1016/j.jenvrad.2009.01.001>

Malczewski D, Dziurowicz M, Kalab Z, Rösnerová M (2021) Natural radioactivity of rocks from the historic Jeroným Mine in the Czech Republic. Environmental Earth Sciences 80(18): 1–11. https://doi.org/10.1007/s12665-021-09944-5

Manisa K, Erdogan M, Usluer A, Cetinkaya H, Isik U, Sahin L, Zedef V (2021) Assessment of natural radioactivity level of soil and water in the region of Çorlu (Turkey). Journal of Radioanalytical and Nuclear Chemistry 329(3): 1213–1221. https://doi.org/10.1007/s10967-021-07906-6

Mantero J, Thomas R, Holm E, Rääf C, Vioque I, Ruiz-Canovas C, García-Tenorio R, Forssell-Aronsson E, Isaksson M (2020) Pit lakes from Southern Sweden: natural radioactivity and elementary characterisation. Scientific Reports 10(1): 1–18. https://doi.org/10.1038/s41598-020-70521-0

Marcon A. E, Navoni J. A, de Oliveira Galvão M. F, Garcia A. C. F. S, do Amaral V. S, Petta R. A, Campos T. F. da, Panosso R, Quinelato A. L, de Medeiros S. R. B (2017) Mutagenic potential assessment associated with human exposure to natural radioactivity. Chemosphere 167: 36–43. https://doi.org/10.1016/j.chemosphere.2016.09.136

Melgar M. J, García M. Á (2021) Natural radioactivity and total K content in wild-growing or cultivated edible mushrooms and soils from Galicia (NW, Spain). Environmental Science and Pollution Research 28(38): 52925–52935. https://doi.org/10.1007/s11356-021-14423-2

Nguyen V. D, Trinh D. H (2022) Natural radioactivity and radiological hazard evaluation in surface soils at the residential area within Ban Gie monazite placer, Nghe An. Journal of Radioanalytical and Nuclear Chemistry 331(2) 769–781. https://doi.org/10.1007/s10967-021-08171-3

Nursapina N. A, Shynybek B. A, Matveyeva I. V, Nazarkulova S. N, Štrok M, Benedik L, Ponomarenko O. I (2022) Effect of mineral fertilisers application on the transfer of natural radionuclides from soil to radish (Raphanus sativus L.). Journal of Environmental Radioactivity, 247(March). <https://doi.org/10.1016/j.jenvrad.2022.106863>

Özden S, Aközcan S (2021) Natural radioactivity measurements and evaluation of radiological hazards in sediment of Aliağa Bay, İzmir (Turkey). Arabian Journal of Geosciences 14(1). https://doi.org/10.1007/s12517-020-06446-9

Pourimani R, Fardad R, Khalili H (2020) Radiological hazard assessment of radionuclides in sediment and water samples of international Meighan Wetland in Arak, Iran. Iranian Journal of Medical Physics 17(2): 107–113. https://doi.org/10.22038/ijmp.2019.39081.1512

Punniyakotti J, Ponnusamy V (2017) Mineralogical role on natural radioactivity content in the intertidal sands of Tamilnadu coast (HBRAs region), India. Journal of Radioanalytical and Nuclear Chemistry 314(2): 949–959. https://doi.org/10.1007/s10967-017-5449-x

Ramasamy V, Paramasivam K, Suresh G, Jose M. T (2014) Function of minerals in the natural radioactivity level of Vaigai River sediments, Tamilnadu, India - Spectroscopical approach. Spectrochimica Acta Part A: Molecular and Biomolecular Spectroscopy 117: 340–350. https://doi.org/10.1016/j.saa.2013.08.022

Ramli A. T, Abdul Rahman A. T, Lee M. H (2003) Statistical prediction of terrestrial gamma radiation dose rate based on geological features and soil types in Kota Tinggi district, Malaysia. Applied Radiation and Isotopes 59(5–6): 393–405. https://doi.org/10.1016/j.apradiso.2003.08.003

Ramli A. T, Sahrone S, Wagiran H (2005) Terrestrial gamma radiation dose study to determine the baseline for environmental radiological health practices in Melaka state, Malaysia. Journal of Radiological Protection 25(4): 435–450. https://doi.org/10.1088/0952-4746/25/4/006

Rani A, Singh S (2005) Natural radioactivity levels in soil samples from some areas of Himachal Pradesh, India using γ-ray spectrometry. Atmospheric Environment 39(34): 6306–6314. https://doi.org/10.1016/j.atmosenv.2005.07.050

Ribeiro F. C. A, Silva J. I. R, Lima E. S. A, do Amaral Sobrinho N. M. B, Perez D. V, Lauria D. C (2018) Natural radioactivity in soils of the state of Rio de Janeiro (Brazil): Radiological characterisation and relationships to geological formation, soil types and soil properties. Journal of Environmental Radioactivity 182: 34–43. https://doi.org/10.1016/j.jenvrad.2017.11.017

Salahel din K (2022) Soil radioactivity levels and radiation exposure to the population in Aswan and Abu Simbel areas, South of Egypt. Physics and Chemistry of the Earth 127, 103179. https://doi.org/10.1016/j.pce.2022.103179

Saleh H, Abu Shayeb M (2014) Natural radioactivity distribution of southern part of Jordan (Ma′an) Soil. Annals of Nuclear Energy 65: 184–189. https://doi.org/10.1016/j.anucene.2013.10.042

Saleh M. A, Ramli A. T, Alajerami Y, Aliyu A. S (2013) Assessment of environmental 226Ra, 232Th and 40K concentrations in the region of elevated radiation background in Segamat District, Johor, Malaysia. Journal of Environmental Radioactivity 124: 130–140. https://doi.org/10.1016/j.jenvrad.2013.04.013

Saleh M. A, Ramli A. T, Alajerami Y, Aliyu A. S, Damoom M (2014) Assessment of health hazard due to natural radioactivity in Kluang District, Johor, Malaysia. Isotopes in Environmental and Health Studies 50(1): 103–113. https://doi.org/10.1080/10256016.2013.821469

Saleh M. A, Ramli A. T, Hamzah K. bin, Alajerami Y, Moharib M, Saeed I (2015) Prediction of terrestrial gamma dose rate based on geological formations and soil types in the Johor State, Malaysia. Journal of Environmental Radioactivity 148: 111–122. https://doi.org/10.1016/j.jenvrad.2015.05.019

Sankaran Pillai G, Chandrasekaran S, Sivasubramanian K, Baskaran R, Venkatraman B (2018) A review on variation of natural radioactivity along the southeast coast of Tamil Nadu for the Past 4 Decades (1974-2016). Radiation Protection Dosimetry 179(2): 125–135. https://doi.org/10.1093/rpd/ncx233

Sanusi M. S. M, Ramli A. T, Gabdo H. T, Garba N. N, Heryanshah A, Wagiran H, Said M. N (2014) Isodose mapping of terrestrial gamma radiation dose rate of Selangor state, Kuala Lumpur and Putrajaya, Malaysia. Journal of Environmental Radioactivity 135: 67–74. https://doi.org/10.1016/j.jenvrad.2014.04.004

Shohda A. M, Draz W. M, Ali F. A, Yassien M. A (2018) Natural radioactivity levels and evaluation of radiological hazards in some Egyptian ornamental stones. Journal of Radiation Research and Applied Sciences 11(4): 323–327. https://doi.org/10.1016/j.jrras.2018.06.002

Shuaibu K. H, Khandaker U. M, Alrefae T, Bradley D. A (2017) Assessment of natural radioactivity and gamma-ray dose in monazite rich black Sand Beach of Penang Island, Malaysia. Marine Pollution Bulletin, 119(1): 423–428. <https://doi.org/10.1016/j.marpolbul.2017.03.026>

Silva C. R. e, Machado D. V, da Silva-Filh E. V. (2019) Determination of the natural radioactivity in the mineral water distributed in the Salutaris Park, Paraíba do Sul, Brazil. Environmental Earth Sciences 78(22): 1–9. https://doi.org/10.1007/s12665-019-8661-x

Szabó Z. Völgyesi P, Nagy H. É, Szabó C, Kis Z, Csorba O (2013) Radioactivity of natural and artificial building materials - a comparative study. Journal of Environmental Radioactivity 118: 64–74. https://doi.org/10.1016/j.jenvrad.2012.11.008

Szkliniarz K, Walencik‐łata A, Kisiel J, Polaczek‐grelik K, Jędrzejczak K, Kasztelan M, Szabelski J, Orzechowski J, Tokarski P, Marszał W, Przybylak M, Fuławka K, Gola S (2021) Characteristics of natural background radiation in the polkowice‐sieroszowice mine, Poland. Energies, 14(14): 1–14. https://doi.org/10.3390/en14144261

Wan Mahmood Z. U, Yii M. W (2012) Marine radioactivity concentration in the Exclusive Economic Zone of Peninsular Malaysia: 226Ra, 228Ra and 228Ra/ 226Ra. Journal of Radioanalytical and Nuclear Chemistry 292(1): 183–192. https://doi.org/10.1007/s10967-011-1413-3

Xu Q, Song C, Chen W, Liu X, & Zhang F (2006) Structure and infrared radiation properties of substituted cordierites. Journal Wuhan University of Technology, Materials Science 21(4): 68–70. https://doi.org/10.1007/BF02841208

Xu S, Zhang G, Dong G, Sun W, Wei D, Li H, Jin Z, Fan Z, & Liu Y (2022) Radiological, geochemical, and environmental assessment in Xuancheng, China: The airborne gamma-ray spectrometric view. Journal of Geochemical Exploration 236, 106980. https://doi.org/10.1016/j.gexplo.2022.106980

Yakovlev E, Druzhinina A, Druzhinin S, Zykov S, & Ivanchenko N (2022) Assessment of physical and chemical properties, health risk of trace metals and quality indices of surface waters of the rivers and lakes of the Kola Peninsula (Murmansk Region, North–West Russia). Environmental Geochemistry and Health 44 (8). https://doi.org/10.1007/s10653-021-01027-5

Yii M. W, & Wan Mahmood Z. U (2011) Radioactivity of plutonium isotopes, 137Cs and their ratio in sediment, seawater and biota from the east coast of Peninsular Malaysia. Journal of Radioanalytical and Nuclear Chemistry, 289(3): 819–833. https://doi.org/10.1007/s10967-011-1149-0
